# Controlling Li Dendritic Growth in Graphite Anodes by Potassium Electrolyte Additives for Li-Ion Batteries

**DOI:** 10.1021/acsami.2c11175

**Published:** 2022-09-12

**Authors:** Sanghamitra Moharana, Geoff West, Marc Walker, Xinjie S. Yan, Melanie Loveridge

**Affiliations:** † Warwick Manufacturing Group (WMG), University of Warwick, Coventry CV4 7AL, U.K.; ‡ Department of Physics, University of Warwick, Coventry CV4 7AL, U.K.; § Impression Technologies Ltd., Unit E Lyons Park, 46 Sayer Drive, Coventry CV5 9PF, U.K.

**Keywords:** fast charging, potassium cation, lithium dendrites, solid electrolyte interphase, lithium fluoride, lithium-ion batteries

## Abstract

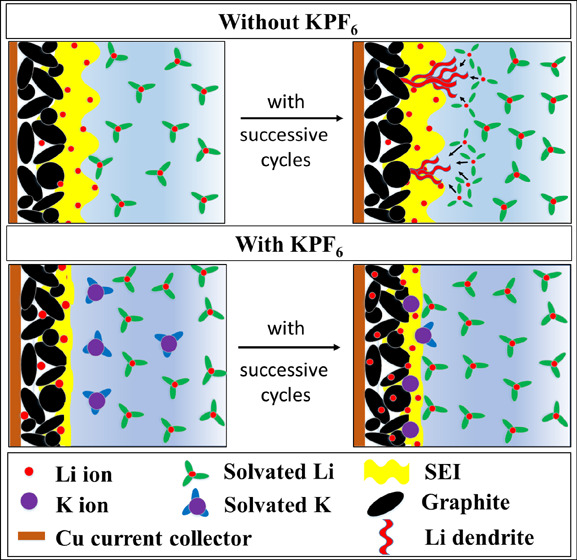

Fast charging promotes Li dendrite formation and its growth on graphite anodes, which affects cell performance in Li-ion batteries (LIBs). This work reports the formation of a robust SEI layer by introducing a KPF_6_ inorganic additive into the electrolyte. An optimal concentration of 0.001 M KPF_6_ effectively inhibits the growth of Li dendrites at 2C charging rates, compared with a commercial electrolyte. Electrolytes containing a KPF_6_ additive are shown here to deliver dual effects to mitigate the growth of dendrites. A thin LiF-rich SEI layer is formed on graphite, which blocks the electron leakage pathways. Additionally, K^+^ resides at defect sites (such as particle boundaries) due to its faster diffusion rate and blocks the incoming Li^+^ and restricts the growth of Li dendrites. The electrolyte with optimum concentration of KPF_6_, i.e., 0.001 M, effectively directs Li^+^ transport through the thin, durable, and low resistance LiF-rich SEI layer. This has implications for fast charging through optimization of the electrode/electrolyte interphase by controlling additive concentrations.

## 1 Introduction

The commercialization of LIBs in the past decade of the 20th century revolutionized energy storage technology.^1^ Since then, LIBs have been extensively used in a variety portable electronic devices such as mobile phones, watches, and laptops. More recently, with global efforts focused on decreasing the use of nonrenewable resources and their emissions, LIBs are now enabling the widespread use of electric vehicles (EVs). In order to efficiently electrify the transportation sector, charging times for vehicles needs to be reduced, which requires faster charging capabilities for present-day LIBs.

Graphite is still used as the predominant anode material in commercial LIBs, with lithium transition metal oxide cathodes such as LiCoO_2_ (LCO), LiNi_*x*_Co_*y*_Al_*z*_O_2_, and LiNi_*x*_Mn_*y*_Co_*z*_O_2_ (NMC), where *z* = 1 – *x* – *y*.^2,3^ When the cell is charged, Li ions from the cathode travel through the electrolyte toward the anode. The electrolyte, which is a combination of linear and cyclic carbonates, with a conducting Li salt, decomposes on the graphite surface and forms an electrode/electrolyte interphase layer called the solid–electrolyte interphase (SEI).^4^ This protective film prevents further decomposition of electrolyte solvents, thus necessitating the need for a stable and intact SEI layer for long-range performance of a LIB cell. Unfortunately, fast charging triggers side reactions such as electrolyte decompositions, resulting in SEI growth on the surface of the graphite. The thicker SEI generates resistance to the Li^+^ intercalation kinetics, which produces heat and leads to a rise in temperature of the cell.^5^ This process further dries out the electrolyte by decomposition, resulting in degradation of the cell. Additionally, metallic lithium deposition on graphite is another major obstacle in fast charging as the Li intercalation potential of graphite is very close to the potential of metallic Li deposition.^5−7^ Therefore, during fast charging, the overpotential drives the electrodeposition process. The deposited metallic Li, being highly active, reacts with electrolyte and forms decomposition products that contribute to SEI growth. This self-accelerated process is problematic as it eventually consumes the electrolyte, as well as the available electrochemically active Li, leading to irreversible capacity loss of the cell. Moreover, the Li plating poses extreme safety issues once it grows in the form of dendrites, when diffusion time becomes limiting.^8^ Electrolyte additive incorporation is one of the most effective and economical approaches used to control the growth of Li dendrites in graphite anodes, thus assisting in enabling faster charging. Although the proposed solid-state electrolytes (SSEs)^9,10^ and solid polymer electrolytes (SPEs)^11^ have appeared to prevent dendritic growth, the respective higher impedance and lower ionic conductivity adversely affect the electrochemical performance of the cell. Hence, selecting an appropriate additive for liquid electrolytes is crucial in constructing an efficient SEI layer in order to prevent Li dendrite formation and growth. The modified SEI layer prevents the nucleation of Li metal through various mechanisms such as (i) formation of adatoms at the hot spot areas,^12^ (ii) repulsion of Li^+^ around the hot spot areas resulting in uniform Li^+^ distribution,^13^ and (iii) homogenization of Li^+^ flux by increasing Li^+^ ionic conductivity.^14^ Recently, a very small concentration of alkali cations such as Cs^+^, Na^+^, Rb^+^ as electrolyte additives have proved to be effective in restricting dendrite growth without hampering any electrochemical properties (such as ionic conductivity and resistance).^15−25^ For instance, potassium is reported to prevent Li dendrite growth by increasing the inorganic components of the SEI layer.^26^ The increased inorganic components in SEI enhances its mechanical strength and Li^+^ ion diffusion, thereby reinforcing its stability against dendritic growth.^27^ Similarly, Zhuang and Zheng et al. investigated various potassium salt additives, which were found to reduce the irreversible Li loss due to K accumulation in the double layer.^28,29^ In addition, K^+^ improved the electrochemical performance by expanding the graphite layers (due to larger K^+^ intercalation) in the very first charge and increasing the Li_2_CO_3_ SEI compound respectively, which reportedly favored the Li^+^ intercalation.^30,31^ Furthermore, enhanced Li^+^ kinetics was confirmed with a K_2_CO_3_ coating on graphite anodes, compared to the Na equivalent.^32^ However, Komaba et al. observed the inferior electrochemical behavior of potassium electrolyte additives compared with Na^+^.^33^ These above contradictory literature studies failed to elucidate the impact of additive concentrations on graphite anode and hence the mechanism behind the Li dendrite growth inhibition, which is the primary focus in this work. Therefore, a K^+^ electrolyte additive is systematically investigated with respect to Li dendrites, in order to have a profound understanding of its impact on graphite anodes. This study attempted to establish the correlation between the concentration of the additive, charging rate, Li deposition, and its inhibition.

In this study, the electrochemical performances of graphite|NMC 622 cell using various concentrations of KPF_6_ containing electrolytes are examined. The incorporation of KPF_6_ additive salt and its impact at various charging rate are evaluated to understand the influence of the additive on the Li deposition and the SEI compositions on graphite anode. The electrochemical and post-mortem studies are performed to determine the optimized electrolyte for graphite|NMC 622 cell upon fast charging.

## 2 Experimental Procedures

### 2.1 Materials

Single side coated artificial graphite (Hitachi MagE3) and LiNi_0.6_Mn_0.2_Co_0.2_O_2_ (Targray NMC 622) electrodes were provided by Argonne’s Cell Analysis, Modeling, and Prototyping (CAMP) Facility, Argonne National Laboratory (ANL), USA. The details of the electrodes are stated in Table S1. A commercial electrolyte consisting of EC (ethylene carbonate):EMC (ethyl methyl carbonate) (3:7 v/v), 1 M LiPF_6_ (lithium hexafluorophosphate), 1 wt % VC (vinylene carbonate) (PuriEL, Soulbrain) was used as a reference electrolyte in this work. In order to investigate the impact of potassium additive, various concentrations of KPF_6_ (potassium hexafluorophosphate) ranging from 0.001 to 0.2 M were used while maintaining the compositions of solvents, Li salt, and VC additive (consistent with that of commercial electrolytes). Battery grade EC, EMC, VC, LiPF_6_ salt, and KPF_6_ salt were purchased from Sigma-Aldrich. Both Li and K salts were dried under vacuum at 60 °C in a Buchi oven to remove the excess moisture before electrolyte formulation. Electrolyte preparation was carried out inside an Mbraun glovebox (O_2_ and H_2_O < 0.5 ppm), and the formulations are tabulated in Table 1. The nomenclature emphasizes the presence of KPF_6_ additive concentration in the electrolyte. It should be noted that E-0M nomenclature is assigned to the commercial electrolyte.

**Table 1:** A List Showing the Formulated Electrolytes Used in This Study

electrolyte nomenclature	formulation
E-0M	1 M LiPF_6_ in EC:EMC (3:7 v/v), 1 wt % VC (commercial electrolyte)
E-0.001M	1 M LiPF_6_ in EC:EMC (3:7 v/v), 1 wt % VC, 0.001 M KPF_6_
E-0.01M	1 M LiPF_6_ in EC:EMC (3:7 v/v), 1 wt % VC, 0.01 M KPF_6_
E-0.1M	1 M LiPF_6_ in EC:EMC (3:7 v/v), 1 wt % VC, 0.1 M KPF_6_
E-0.15M	1 M LiPF_6_ in EC:EMC (3:7 v/v), 1 wt % VC, 0.15 M KPF_6_
E-0.2M	1 M LiPF_6_ in EC:EMC (3:7 v/v), 1 wt % VC, 0.2 M KPF_6_

### 2.2 Electrochemical Testing

Graphite and NMC 622 electrode sheets were cut into disks of 15 mm ⌀ and 14.8 mm ⌀, respectively, and vacuum-dried in a Buchi oven at 120 °C, to remove excess moisture prior to assembly. The separator used for the coin cell assembly was PP–PE–PP microporous trilayer membrane (Celgard 2325) and was cut into a larger size, i.e., 19 mm ⌀, to avoid physical contact between the electrodes. The volume of electrolyte used for graphite|NMC 622 full cell was 100 μL. The graphite|NMC 622 full cells were assembled into Hohsen 2032-type coin cells inside the argon-filled Mbraun glovebox (O_2_ and H_2_O < 0.5 ppm). Electrochemical testing was performed on a BCS BT-Lab potentiostat at ambient temperature. All the coin cells were initially cycled twice at C/20 rate (∼0.14 mA) for formation, followed by C/5 (∼0.55 mA) and C/2 (∼1.38 mA) slow charging to establish the baseline for the full cell. Similar to slow charging, the cells were formed twice at C/20 rate followed by cycling at various C-rates ranging from 1C to 3C, up to 100 cycles at ambient temperature. The full cell charging was carried out in constant current–current voltage (CCCV) mode, whereas the discharge was performed in CC mode. The voltage range for charging was 4.2–3 V.

An ECC-PAT-core EL-cell with three-electrode setup was used to investigate the electrochemical behavior of each electrode distinctively, which was not possible with a two-electrode coin cell setup. The electrodes were cut into 18 mm ⌀ disks and assembled into EL-cell inside the Mbraun glovebox (O_2_ and H_2_O < 0.5 ppm). The expanded view of EL-cell components and its assembly is presented in Figure S1. An insulation sleeve of Whatman borosilicate glass fiber of 260 μm thickness in-built separator and a Li ring as reference electrode were used for three-electrode experiments. The EL-cells were cycled at various C-rates in galvanostatic mode ranging from C/5 to 3C to examine the electrochemical performance of both graphite and NMC 622 electrodes with respect to a Li reference electrode.

Electrochemical impedance spectroscopy (EIS) was performed with three-electrode EL-cell using a Biologic VMP3 potentiostat. The EIS spectra were recorded to investigate the effect of additive concentrations on the impedance in a full cell (graphite|NMC 622) along with the half cells (graphite|Li and NMC 622|Li). EIS experiments were conducted in the frequency range of 500 kHz to 10 mHz with a voltage amplitude of 10 mV. All EIS spectra were obtained at 50% state-of-charge (SoC) after the first cycle and at each 10 cycle intervals until 100 cycles. A relaxation time of 30 min was maintained to achieve the equilibrium state prior to EIS measurement. Following this, the EIS spectra were fitted with a simplified Randles circuit using ZView software.

### 2.3 Post-Mortem Characterization

The coin cells were disassembled after 100 cycles in a fully discharged condition (3 V) in an Mbraun glovebox to reduce moisture and oxygen contamination. Afterward, the cycled graphite electrodes were carefully extracted and dried inside the glovebox for electrolyte evaporation. SEM was performed with a field-emission scanning electron microscope (FE-SEM) (Sigma, Zeiss) equipped with an energy dispersive X-ray spectrometer (Xmax^N^ 80, Oxford Instruments). This was used to investigate the morphological evolution of cycled graphite electrodes upon additive incorporation as well as fast charging. In order to preserve the microstructure, the cycled electrodes were transferred to the SEM chamber with a specially designed airless transfer device (Kammrath & Weiss). The SEM images were collected using an in-lens detector with an accelerating voltage of 10 kV and aperture size of 60 μm. EDX was performed on cycled graphite anode for elemental study.

X-ray photoelectron spectroscopy (XPS), secondary ion mass spectroscopy (SIMS), and Raman spectroscopy were performed to study the chemical composition of solid electrolyte interphase (SEI) present on cycled graphite with respect to additive concentration in the electrolyte. XPS was carried out using an Axis Ultra DLD spectrometer (Kratos Analytical Ltd.) with a monochromatic Al Kα X-ray (1486.7 eV) source for excitation. The core level XPS spectra were recorded at room temperature at a takeoff angle of 90° with respect to surface parallel and with a pass energy of 20 eV (resolution ∼0.4 eV). The work function and the binding energy scale of the spectrometer were calibrated using Fermi edge and 3d_5/2_ peak, recorded from a polycrystalline Ag sample prior to the experiments. The cycled graphite electrodes were mounted on a 15 mm diameter Cu stub and transferred to the XPS chamber through an airless transfer device. In order to prevent surface charging effects, the sample surface was flooded with a beam of low energy electrons throughout the experiments, which generates the need for recalibration of the binding energy scale. Therefore, the recorded XPS spectra were modeled by referencing the C 1s spectrum at a binding energy of 285.0 eV. The core level spectra were modeled using the CasaXPS software package, employing Shirley backgrounds and mixed Gaussian–Lorentzian (Vigot) line shapes.

SIMS measurements were carried out in FEI Scios dual beam scanning electron/focused ion beam microscope equipped with a quadrupole mass analyzer (EQS, Hiden Analytical). The cycled electrode samples were placed in the airless transfer device and was guided to the microscope stage by interfacing it with the microscope chamber through an opening gate valve. The sample loaded microscope stage was then set to the eucentric height of 7 mm. Afterward, the sample stage was tilted to 52° for operation in order to make the sample surface normal to the ion beam direction. SIMS measurements were carried out under high vacuum conditions to avoid the collision of background gas molecules with secondary ions ejected from the sample. The mass spectra were obtained by sputtering Ga^+^ ions to the sample at an accelerating voltage of 30 kV and beam current of 0.5 nA. An in-built software named MASsoft Professional 7 was used for recording and analyzing the data. Both positive and negative profiles were recorded on the surface of the cycled graphite electrode.

Raman spectroscopy was performed in a Renishaw Invia micro-Raman spectrometer, using a DXR microscope and a diode-pumped solid-state laser (RL523C50), with a laser excitation wavelength of 532 nm at a laser power of 5 mW. The Raman spectrum was obtained by single point scanning using OMNICxi software.

## 3 Results and Discussion

### 3.1 Electrochemical Characterization

**Figure 1:**
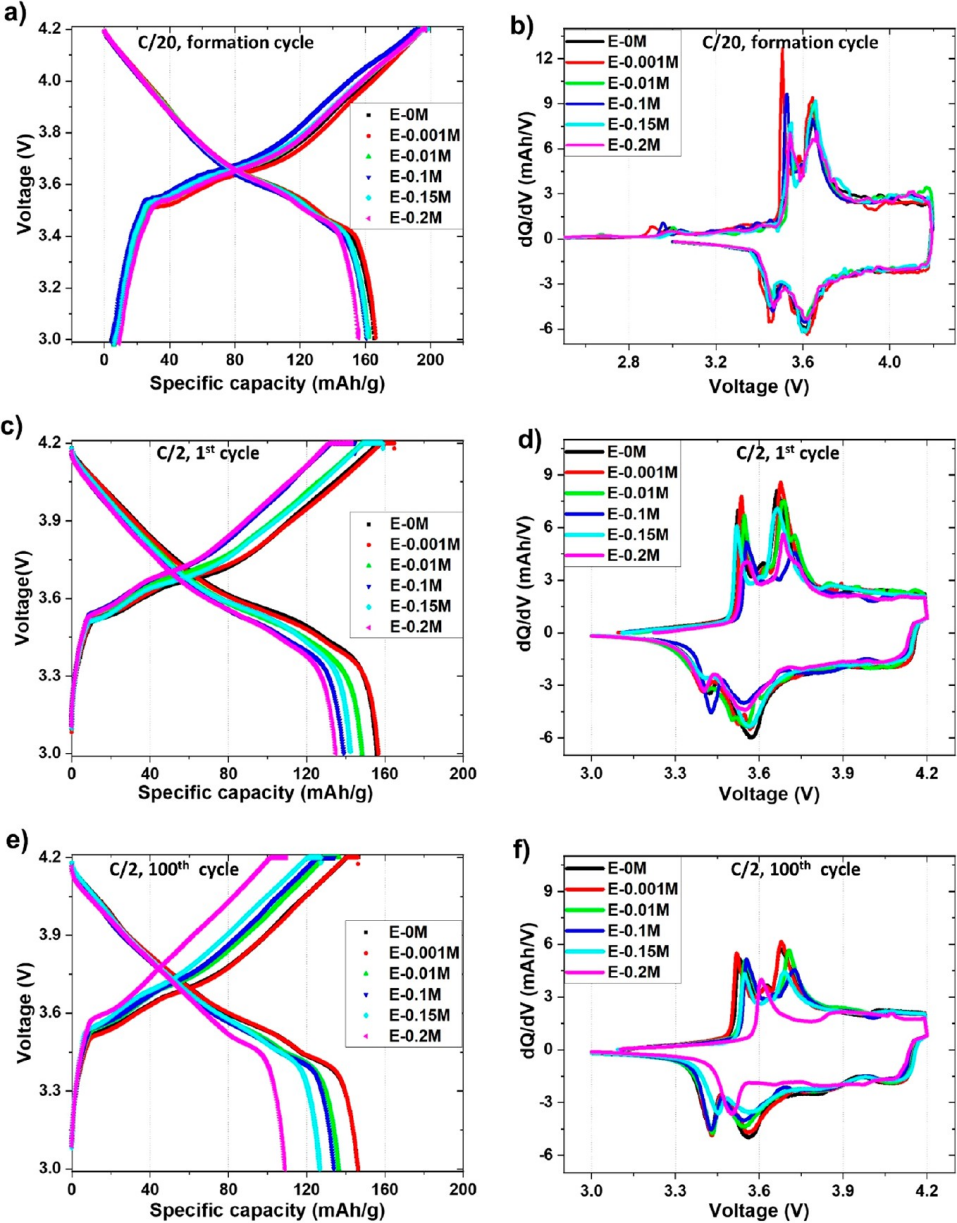
Voltage vs capacity profile of different electrolytes of (a) formation cycles at C/20, (c) 1st cycle, (e) 100th cycle at C/2 slow charging and corresponding d*Q*/d*V* vs *V* plots (b, d, f).

Figure 1 shows the charge/discharge voltage profile and the corresponding incremental capacity plot (d*Q*/d*V* vs *V*) of MagE3 graphite|NMC 622 full cell comprising different modified electrolytes. Figure 1a shows the voltage vs capacity plot where the maximum specific discharged capacities are relatively close to each other i.e., ∼166 mAh/g, ∼162 mAh/g, ∼161 mAh/g, ∼161 mAh/g, and ∼156 mAh/g, respectively. However, a decreasing trend is observed with increase in KPF_6_ concentration from 0.001 M to 0.2 M. The full cell was formed at a slow current rate of C/20 (∼0.14 mA) to produce a stable and protective SEI layer on graphite surface through electrolyte decomposition, shown as broad and small peaks at ∼2.6–2.95 V ^34^ in Figure 1b. In addition, four sharp and distinct peaks, i.e., two oxidation peaks at ∼3.6 V and 3.7 V and two corresponding reduction peaks at ∼3.45 V and 3.6 V, are observed. The oxidation peak at ∼3.6 V is attributed to Li-intercalation into graphite layers upon charging of the full cell.^34^ Another oxidation peak detected at ∼3.7 V is attributed to the phase transition of NMC from hexagonal-1 (H1) to monoclinic (M).^34^ Furthermore, a small and broad oxidation peak observed at ∼4.1 V (Figure 1b) corresponds to the phase transition of NMC from monoclinic (M) to hexagonal (H2) phase. The hexagonal H2 phase has different lattice parameters compared to the hexagonal H1 phase.^35^

Following formation, graphite|NMC 622 full cells with modified electrolytes were cycled at slow C-rate of C/2 (∼1.38 mA) up to 100 cycles. The slow cycling performance at C/2 is presented in order to establish the baseline for comparison with faster-cycling conditions. Figure 1c shows the first cycle voltage vs capacity profile at C/2, where the discharge capacities obtained by electrolytes E-0M and E-0.001M are almost identical i.e., ∼156 mAh/g. However, the incremental amount of KPF_6_ additive is detrimental to the cell’s performance, as a consequence of increased polarization. The shifting of peak positions (oxidation peaks toward right and reduction peaks toward left) in Figure 1d denotes the internal resistance rise with additive amount, especially for 0.2 M concentration of KPF_6_. In Figure 1e, superior discharge capacities are achieved by both E-0M and E-0.001M electrolyte (∼146 mAh/g) even after the 100th cycle compared to ∼108 mAh/g capacity obtained by E-0.2M electrolyte. This is due to the internal resistance build-up due to the obstruction in Li^+^ ions by larger sized K^+^ ions^36,37^ as the amount of additive increases. It should be noted that the full cell cycled with E-0.2M electrolyte shows only one oxidation peak at ∼3.6 V in contrast to the two peaks recorded for the rest of the electrolytes in Figure 1f. Li^+^ intercalation into the graphite layers is suspected to be associated with this single oxidation peak of E-0.2M electrolyte.

**Figure 2:**
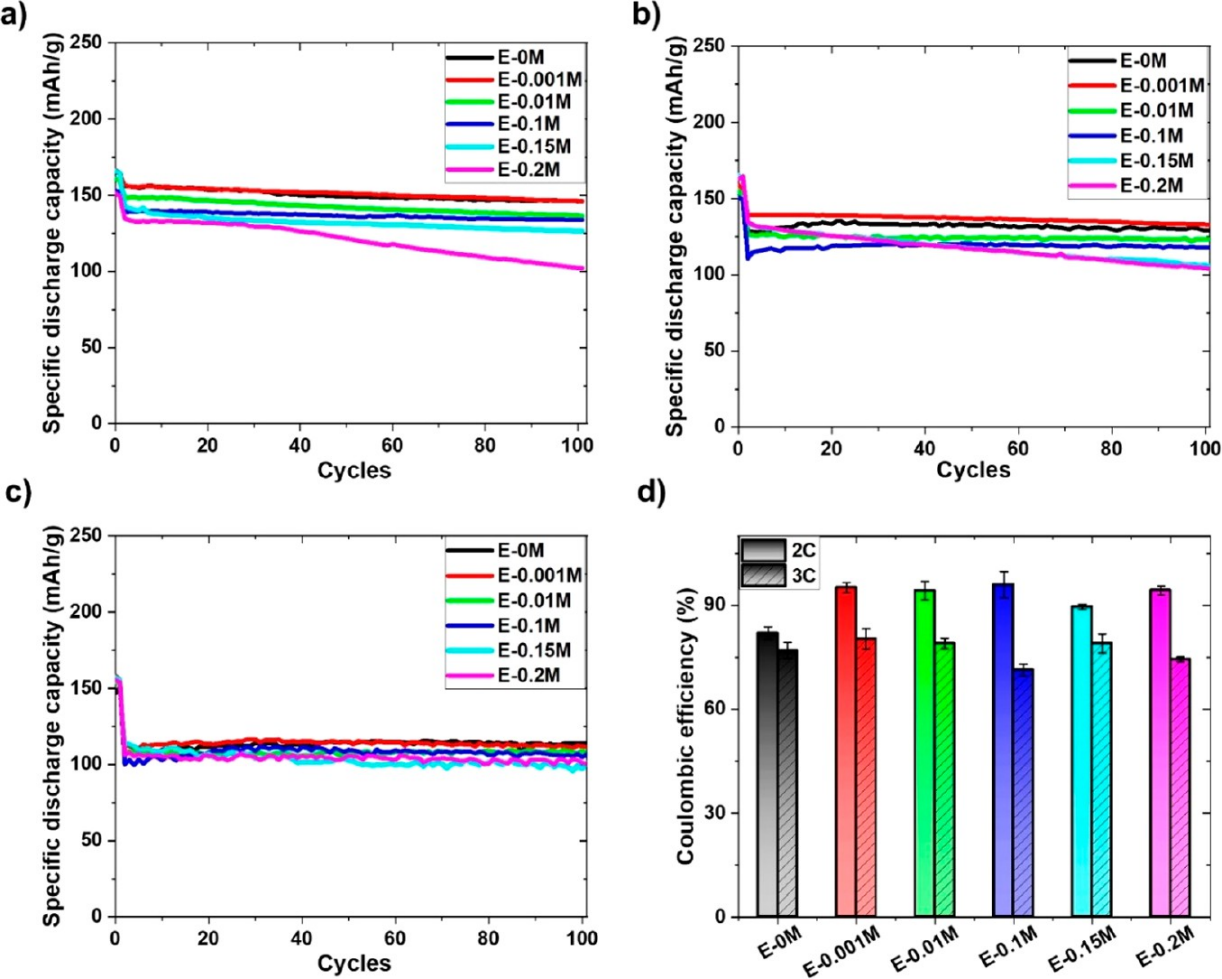
Cycling performance of graphite|NMC 622 full cell at (a) C/2 (∼1.38 mA), (b) 2C (∼5.6 mA), and (c) 3C (∼8.4 mA), (d) Initial Coulombic efficiency comparison at 2C and 3C rate with different modified electrolytes.

For fast cycling, the full cells were cycled at various C-rates ranging from 1C to 3C rate. It is observed that the cells with E-0M and E-0.001M electrolytes demonstrate superior discharge capacities compared with the other modified electrolytes (Figures 2a–c and S2). Furthermore, the specific discharge capacities decrease with increasing amounts of KPF_6_ irrespective of the C-rates. For instance, the capacities achieved by graphite|NMC 622 cells using E-0.001M and E-0.2M electrolytes at C/2 are ∼157 mAh/g and ∼135 mAh/g respectively to begin with, decreasing to ∼146 mAh/g and ∼102 mAh/g by the 100th cycle. The reason behind this could be the hindrance of Li^+^ movement due to the presence of K^+^ with increasing KPF_6_ concentration, affecting the cycling performance adversely. The superior first cycle Coulombic efficiencies (CEs) for E-0.001M electrolyte at 2C and 3C, presented in Figure 2d, suggest the reduced electrolyte decomposition during SEI formation, indicating the decreased irreversible capacity loss. It is noticed that the first cycle CEs at 2C rate are higher for all the modified electrolytes, which have KPF_6_ as electrolyte additive, compared to E-0M commercial electrolyte. The increased CEs for modified electrolytes could be due to the formation of stabilized SEI layer by K additive incorporation that suppresses the parasitic reaction rates (Figure S3) and the corresponding irreversible capacity loss. Among all, E-0.001M electrolyte denotes highest CEs, indicating decreased electrolyte decomposition, parasitic reaction rate, and therefore irreversible capacity loss, for graphite|NMC 622 full cell upon fast charging of 2C. However, the parasitic reaction rates for 3C rate are not significantly improved in additive-based electrolytes with increased KPF_6_ concentrations (0.1–0.2 M). This is suspected due to the deposition of Li or/and K metal, which is investigated and elaborately discussed in a later section.

**Figure 3:**
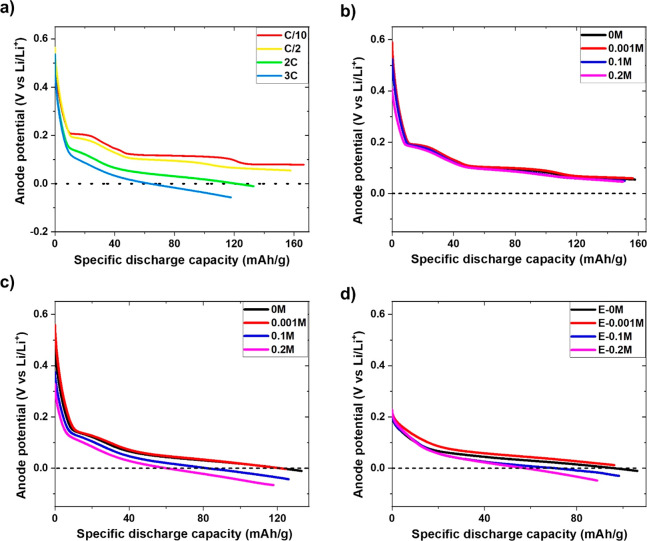
Three-electrode EL-cell (graphite|Li∥NMC 622 | Li) at slow and fast cycling: Anode potential vs specific discharge capacity (a) using E-0M at various charging rate, (b) at C/2 1st cycle (c) at 2C 1st cycle, and (d) at 2C 100th cycle.

In order to better understand the impact of KPF_6_ on graphite anodes, three-electrode EL-cells are assembled using NMC 622 as the working electrode (WE), graphite as the counter electrode (CE), and Li ring as the reference electrode (RE). The anode potential (graphite|Li) using E-0M commercial electrolyte at various charging rate is presented in Figure 3a for reference. It is observed that the anode voltage profile exhibits distinct stages at very slow rate of C/10 (∼0.28 mA). Upon lithiation of graphite, a phase transition from liquid-like phase (stage 1L) to a dense phase called stage 1 (LiC_6_) occurs, giving rise to theoretical capacity of 372 mAh/g.^38,39^ The stages start to disappear as the C-rate increases to 3C. The reason behind this is the relatively lower solid-state diffusion coefficient of Li into graphite in the dense phases compared to the liquid-like phases.^40^ In addition to that, the lack of time available at higher current densities controls the diffusion of Li inside the graphite layers, causing the stages to disappear. It should be noted that the anode potential drops below 0 V vs Li/Li^+^ at 2C rate, indicating metallic Li formation and deposition on the graphite surface. The anode potential drops more quickly to 0 V vs Li/Li^+^ as the current density increases to 3C, demonstrating the earlier deposition and growth of Li metal. Figure 3b shows the anode potential using the modified electrolytes. Three modified electrolytes, i.e., E-0.001M, E-0.1M, and E-0.2M showing superior, intermediate, and inferior performances, were deliberately selected for the three-electrode study. In Figure 3b, there is no visible change observed in the voltage profile at the C/2 rate. In Figure 3c, as the C-rate changes to 2C, the anode potentials drop to 0 V vs Li/Li^+^ for E-0.1M, E-0.2M, and E-0M and remain negative even at the 100th cycle (Figure 3d). However, the profile is slightly different in the case of the E-0.001M electrolyte. The anode potential obtained from the E-0.001M in the first cycle is −0.002 V in contrast to −0.01 V of E-0M. This reveals that the graphite anode potential is influenced by KPF_6_ additive incorporation, henceforth Li metal deposition on graphite surface. This shifting of anode potential toward a less negative value, i.e., from −0.01 V to −0.002 V, occurs by incorporation of only 0.001 M KPF_6_ into the electrolyte. The potential shift signifies the extent the Li deposition is declined in the case of E-0.001 M electrolyte, as the graphite potential moves closer to 0 V vs Li/Li^+^. This proves to be the beneficial effect of KPF_6_ electrolyte additive with regards to Li deposition. The potential profile of E-0.001M at 100th cycle is also examined and found to be 0.01 V (positive) unlike the rest of the electrolytes used (including commercial E-0M electrolyte). This positive graphite potential at 100th cycle implies that Li deposition is restricted in the case of the E-0.001M electrolyte. Further detailed investigations were carried out to interpret this observed behavior.

According to the Nernst equation, the deposition potential for potassium with respect to Li/Li^+^ is as follows:
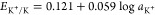
1Equation 1 shows the potassium deposition potential at room temperature.^33^ The detailed derivation is provided in Supporting Information. The calculated potassium deposition potentials for all the modified electrolytes are presented in Table 2.

**Table 2:** Potassium Deposition Potential with Respect to Additive Concentrations in the Electrolyte

concentration of KPF_6_ additive (M)	potassium deposition potential on graphite anode (V)
0.001	–0.056
0.01	0.003
0.1	0.062
0.15	0.072
0.2	0.08

Table 2 denotes the potential at which potassium deposition starts on the graphite surface. Potassium is deposited in the form of metal whenever the graphite anode reaches these potentials. The graphite anode reaches a maximum potential of −0.002 V, −0.04 V, and −0.06 V in the very first cycle for E-0.001M, E-0.1M, and E-0.2M, respectively, already shown in Figure 3c. This signifies metallic potassium deposition for all the modified electrolytes except E-0.001M. It should be noted that the E-0M commercial electrolyte does not contain KPF_6_ additive; thus no potassium deposition is seen. Similarly, the graphite potential is negative for E-0.1M and E-0.2M at the 100th cycle, indicating continuous potassium as well as lithium deposition on the graphite surface. The formation of metallic potassium and its growth implies that the effectiveness of KPF_6_ additive is reduced upon successive cycles. However, the potassium deposition potential is not reached for E-0.001M electrolyte, meaning it continues to restrict Li deposition with further cycling, as shown in Figure 3d.

### 3.2 Post-Mortem Characterization

#### 3.2.1 Morphology Evolution of Graphite after Cycling

**Figure 4:**
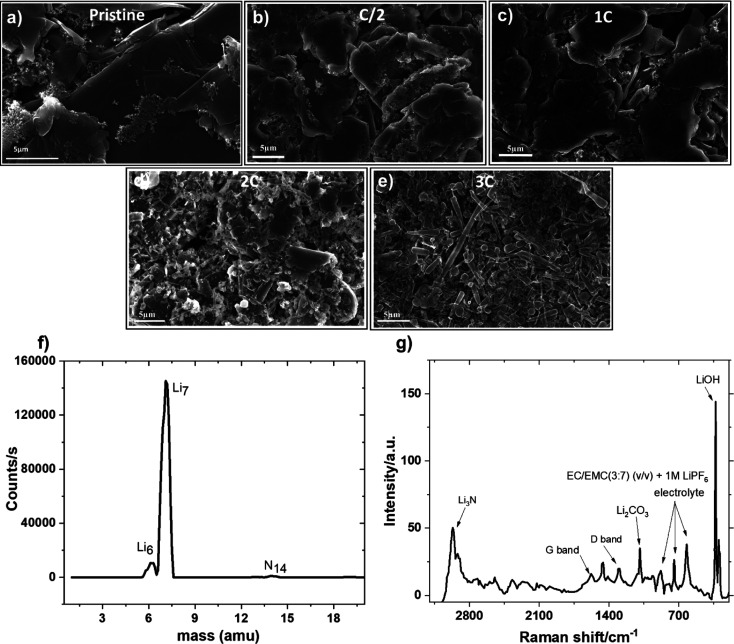
(a–e) SEM images of graphite anode morphologies cycled at different C-rates after 100 cycles using E-0M commercial electrolyte. (f) Positive ion SIMS spectra and (g) Raman spectra of dendrites present on the graphite anode.

Following the cycling of graphite|NMC 622 full cells, the cycled graphite anodes were collected for post-mortem characterization. Figure 4 shows SEM micrographs of the surface morphology of the graphite anodes with different C-rates using the E-0M electrolyte. The morphologies of graphite cycled at C/2 and 1C rate (Figure 4b,c) are similar to that of the pristine graphite anode (Figure 4a) but then changes significantly as the C-rates increase further. In Figure 4d, dendrite-like Li deposition starts at the edge of the graphite flakes at a rate of 2C. This supports the electrochemical result shown in Figure 3a. Defect sites such as edges of the particles and cracks have high energies and hence are prone to Li deposition primarily.^41^ Once the deposition starts, Li attracts other incoming Li atoms to deposit and grow upon further cycling.^42^ As the C-rate rises to 3C, the thickness along with the length of Li dendrites increases. Sometimes the cluster of Li dendrites entirely covers the surface of the graphite anode, shown in the Figure 4e. It is observed that Li dendrites change their direction through kink formation, which depends on the crystallographic plane, direction, and nature of graphite material. The chemical composition of the deposits is examined by SIMS and Raman spectroscopy, which demonstrates that the deposits are of Li metal (Figure 4f,g). SIMS spectra identifies the elemental Li, i.e., ^7^Li, along with its isotope ^6^Li on dendritic deposit. In Figure 4g, the sharp peak at 330 cm^–1^ is caused by Raman shift of OH stretching in LiOH compounds,^43^ which is an SEI component. Additionally, Li dendrites could react with the moisture (eq 2) while transferring the sample into the chamber without any airless device and giving rise to LiOH as a reaction product. Furthermore, a broad peak at 2800–3000 cm^–1^ is observed, which corresponds to Li_3_N.^43^ Li_3_N is formed only when atmospheric nitrogen reacts with lithium in metallic form (eq 3) and confirms the presence of metallic Li on the graphite surface. Moreover, symmetric stretching vibration of Li_2_CO_3_ ^43,44^ and vibrational peaks of EC,^45^ EMC,^46^ LiPF_6_^45^ are also detected. G band (1580 cm^–1^) and D band (1360 cm^–1^) of graphite^44^ having smaller intensities are also identified as graphite becomes fully covered with Li dendrites.
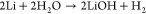
2
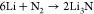
3

**Figure 5:**
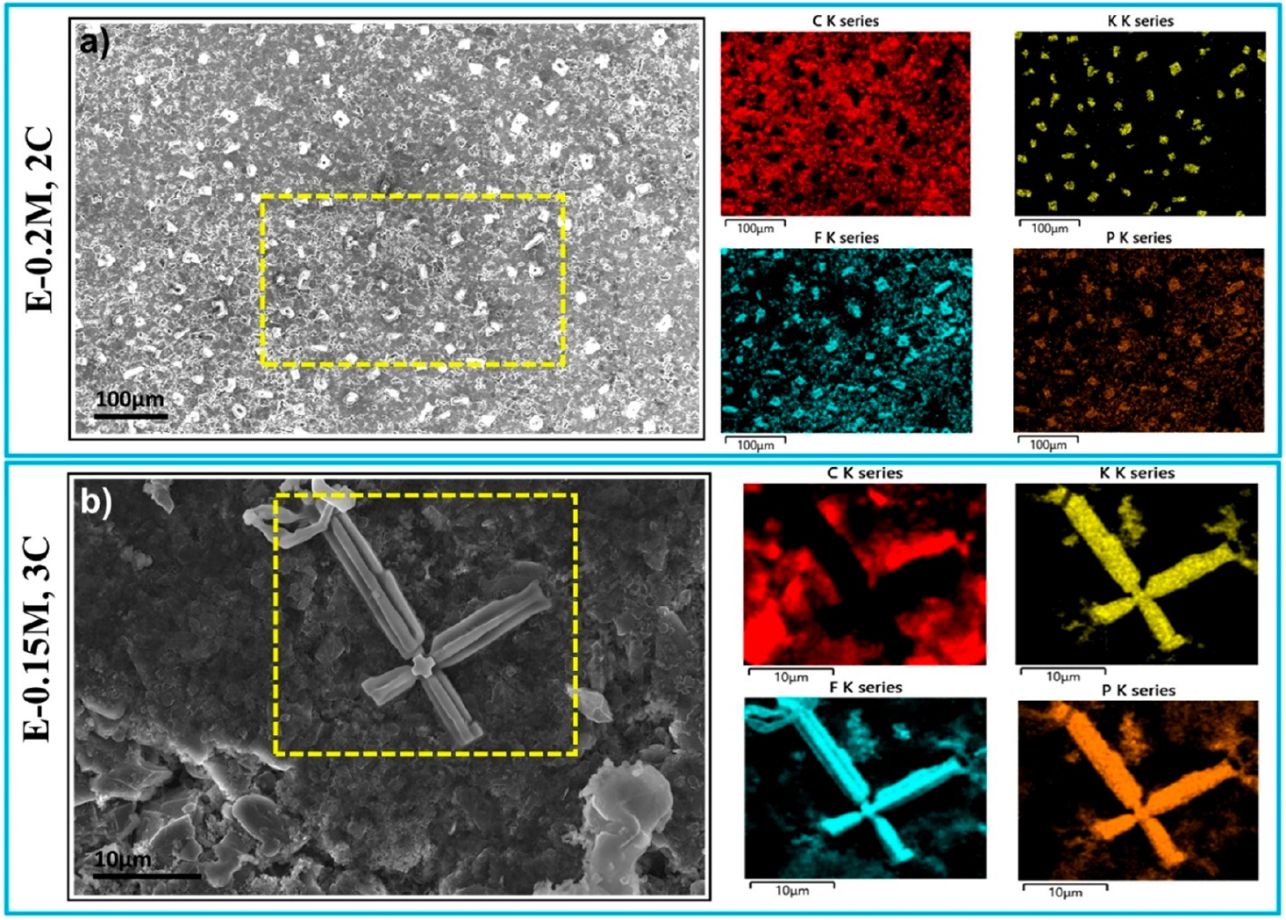
SEM and its corresponding EDX mapping of cycled graphite with E-0.15M and E-0.2M electrolytes presenting K dendrites.

In Figure 5, graphite anodes cycled with E-0.15M and E-0.2M electrolytes experience deposits with morphology different from that of Figure 4. Figure 5a demonstrates the deposits are almost everywhere on the graphite surface (∼300 deposits per mm^2^, calculated using ImageJ software). Further examination by EDX mapping shows three major components, e.g., K, F, and P. The presence of K and absence of C (clearly evident in Figure 5b) ensure that the deposits are of potassium-containing compounds. Although the intensity of F and P appears to be higher on the deposits, the ratio of F/P is almost identical on both deposited and nondeposited areas. This confirms that the deposits are K metal. The presence of potassium dendrites with respect to additive concentration and C-rate is presented in Figure S4. Thus, KPF_6_ additive in E-0.15M and E-0.2M electrolytes fails to control the growth of Li dendrites by producing its own K dendrite, leading to the inferior performance of the cell. It is observed that K dendrites have multiple branches coming out the principle arm in all possible directions (Figure S4d), resembling classical dendritic morphology during the solidification of metals.^47^ Moreover, the different microstructure of K dendrites compared to Li dendrites is due to inherent material properties such as crystallographic planes and directions of individual metals.^48,49^

**Figure 6:**
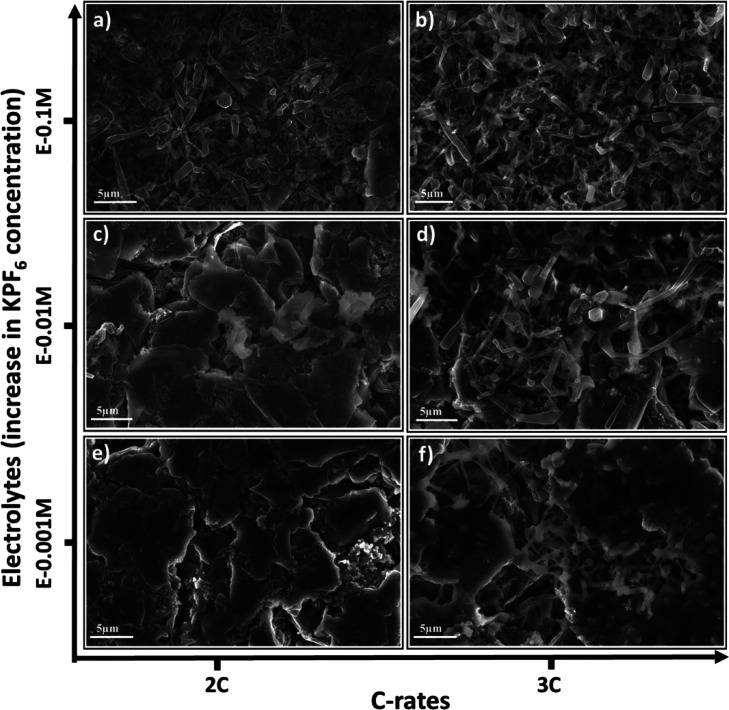
SEM images of cycled graphite anode morphologies using E-0.1M, E-0.01M, and E-0.001M electrolytes at (a, c, e) 2C and (b, d, f) 3C rates.

Figure 6 illustrates SEM images arranged in *X*–*Y* planes, where the X axis represents the charging rate and the *Y* axis is allocated to KPF_6_ additive concentration in the electrolyte. Li dendrites are present on the cycled graphite anodes at 3C rate irrespective of the concentration of the additive, shown in Figure 6b,d,f. However, dendritic portion appears to be reduced with regard to the decrease in KPF_6_ amount in electrolyte at 2C charging rate (Figure 6a,c,e). Ultimately, Li dendrites are seemingly inhibited with 0.001 M KPF_6_ concentration at 2C rate, as demonstrated in Figure 6e. This post-mortem study aligns with the electrochemical result in Figure 3d, where an anode potential of 0.01 V (positive) was obtained at the 100th cycle. Large area imaging was produced at different locations of the sample, presented in Figure S5.

Moreover, the effectiveness of the KPF_6_ additive is diminished at higher concentrations and charging rates.

#### 3.2.2 Mechanism of Li Dendrite Control by Optimized Electrolyte Composition

**Figure 7:**
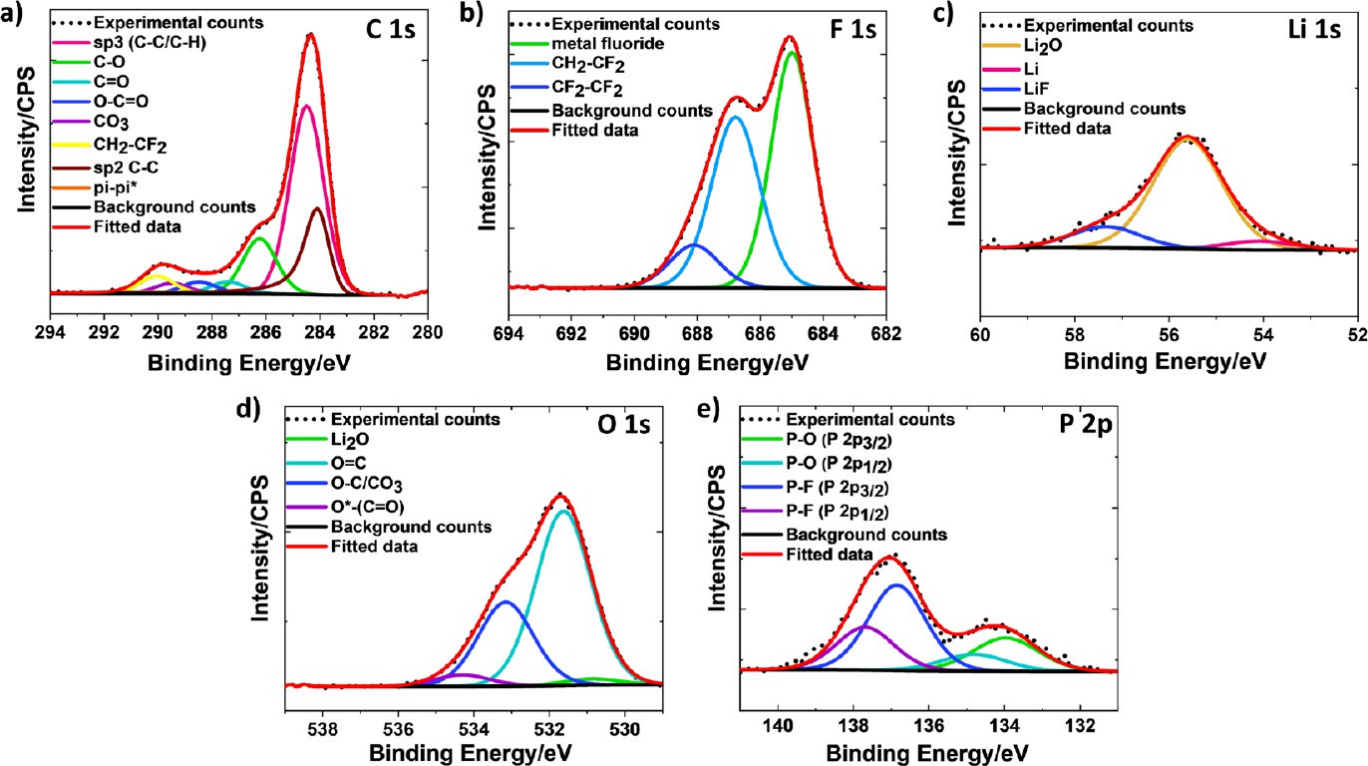
XPS (a) C 1s, (b) F 1s, (c) Li 1s, (d) O 1s, (e) P 2p spectra of graphite anode cycled using E-0.001M electrolyte at 2C rate.

The chemical evolution of SEI layers formed on the graphite anodes using modified electrolytes was observed by XPS. Figure 7 shows the XPS spectra of graphite cycled with E-0.001M electrolyte at 2C. Deconvolution of the C 1s spectrum (Figure 7a) features one principal peak corresponding to sp^3^ carbon. Along with that, C–O (∼286.4 eV), C=O (∼287.6 eV), O=C–O (∼288.6 eV), CO_3_ (∼289.7 eV) peaks are observed, which are the result of the decomposition of the solvents, i.e., EC ([CH_2_O)_2_CO] and EMC [C_2_H_5_OCOOCH_3_] present in the electrolyte.^34^ In addition, a CH_2_–CF_2_ peak (∼290.2 eV) originates from PVDF binder present in graphite electrode coating^34^ (Figure 7a). Furthermore, another prominent peak, due to sp^2^ C–C bonding, is observed at a binding energy of ∼284.3 eV, along with the π–π* shakeup feature at a binding energy of ∼290.7 eV.^50^ The presence of CH_2_–CF_2_ peak indicates that the SEI film is thin enough to allow the photoelectrons ejected from the binder to travel through the SEI layer into the vacuum in order to reach the detector. The presence of CH_2_–CF_2_ and its fluorinated CF_2_–CF_2_ peak^34^ are demonstrated in the F 1s spectrum, shown in Figure 7b. The prominent peak in the F 1s spectrum is at ∼685.0 eV, assigned to metal fluoride (LiF) present in the SEI film. Three components such as Li (∼54 eV), Li_2_O (∼55.6 eV), and LiF (∼57.5 eV) contribute to Li 1s spectrum,^51^ in Figure 7c. The product of electrolyte decomposition such as C=O (∼531.6 eV), C–O/CO_3_ (∼533.2 eV)_,_ O*–(C=O) (∼534.3 eV) together with a small peak of Li_2_O (∼530.8 eV) is observed in O 1s spectrum, shown in Figure 7d. In Figure 7e, the P 2p spectrum gives rise to a doublet peak due to spin–orbit split coupling. The deconvolution of the P 2p spectrum was performed by considering 2p_1/2_ and 2p_3/2_ spin orbital components with constrained specific area ratio and a peak separation value of 0.84 eV.^52,53^ The lower binding energy doublet is attributed to phosphate, i.e., Li_*x*_PO_*y*_F_*x*_ from LiPF_6_ degradation, whereas the higher binding energy doublet is assigned to P–F bonding of LiPF_6_ or Li_*x*_PF_*y*_.^54,55^ XPS spectra of the graphite anode cycled with E-0.1M at 2C rate is displayed in Figure S6, in which the deconvolution of F 1s, Li 1s, and P 2p spectra depicts the components similar to Figure 7. Comparing the XPS spectra of E-0.1M (Figure S6) with E-0.001M (Figure 7), a small C 1s component is detected at lower binding energy of ∼282.5 eV, which signifies the reaction of C with a metal. The component is unlikely to be lithium carbide (Li_2_C_2_), which is implausible to form in LIBs under any condition.^56^ Therefore, carbide-like lithium acetylide (Li–C≡C–X) is highly likely to form upon fast charging of carbon materials.^57−59^ Acetylide species have been reported to be formed when metallic Li reacts with organic and inorganic compounds of the SEI layer.^56,59,60^ The presence of a lithium acetylide (Li–C≡C–X) component in C 1s XPS spectra in E-0.1M electrolyte signifies the presence of metallic Li on the graphite anode. Similarly, its absence signifies the inhibition of metallic Li deposition in E-0.001M electrolyte. This supports the positive anode potential monitored at the 100th cycle in Figure 3d. Additionally, K 2p_3/2_ and K 2p_1/2_ peaks are also identified toward the end of the C 1s spectrum (Figure S6a), which was not observed in E-0.001M’s XPS spectra (Figure 7). The reason for this is the low concentration of additive (0.001 M KPF_6_) present in the electrolyte, which is above the detection limit of XPS.^61^ XRF detects ∼200 ppm potassium present on the graphite anode cycled with E-0.001M electrolyte, which is shown in Table S2 in Supporting Information.

**Figure 8:**
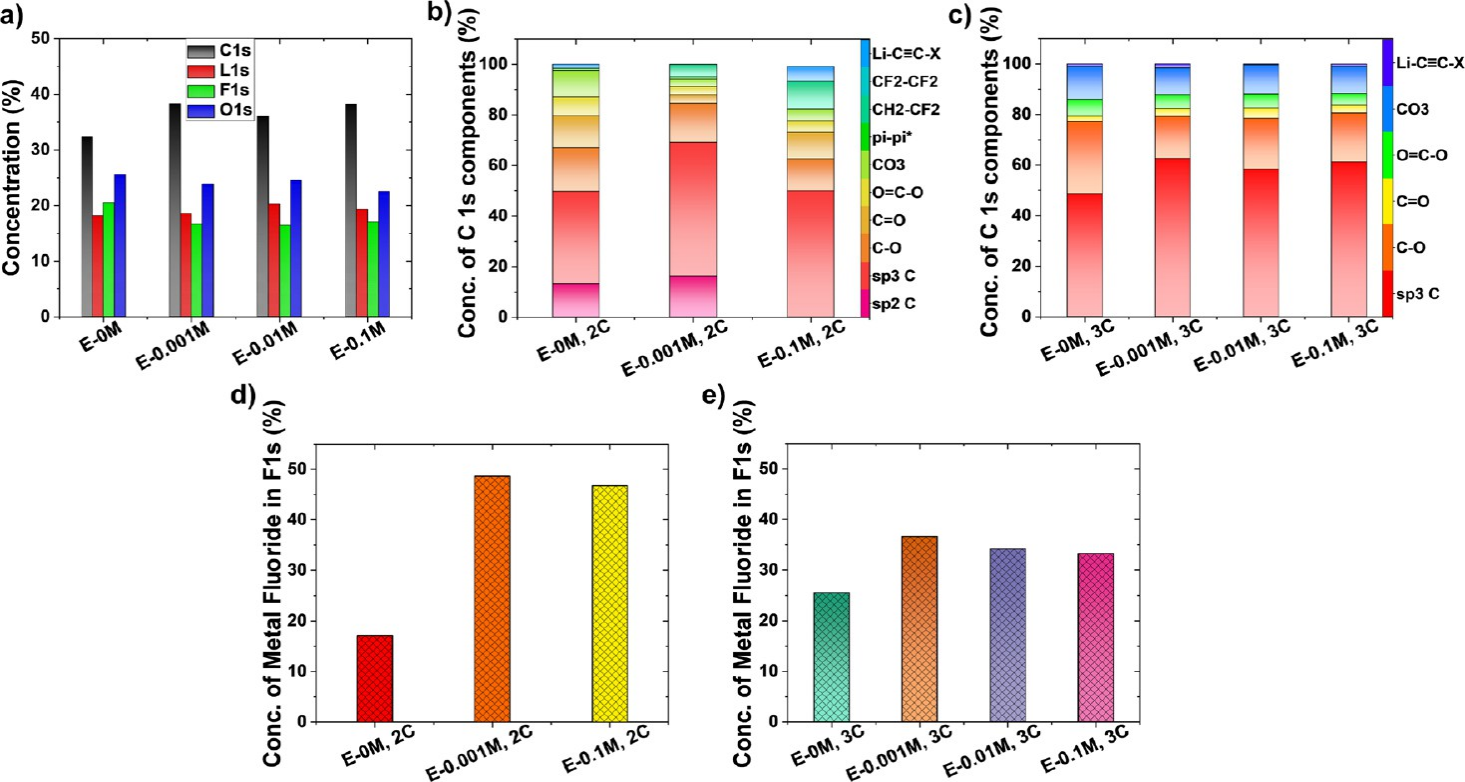
(a) Comparison of percentage concentration of elements detected. C 1s components for graphite in E-0M, E-0.001M, E-0.01M, E-0.1M at (b) 2C and (c) 3C rates. Metal fluoride concentration percentage at (d) 2C and (e) 3C rates.

In order to fully understand the mechanism behind Li dendrite inhibition, deconvoluted XPS spectra are analyzed further. Figure 8a designates the percentage concentration of different elements obtained through the deconvolution of XPS spectra. The increased C 1s and decreased F 1s concentration of modified electrolytes signify thinner SEI film on the graphite surface compared to E-0M electrolyte due to the following reasons: (i) higher C 1s counts that imply reduced attenuation of the photoelectron yield from the graphite anode and (ii) the lower concentration percentages of F 1s (LiF at ∼685 eV), O 1s (Li_2_O at ∼530.8 eV, C=O at ∼531.6 eV, C–O/CO_3_ at ∼533.2 eV, O*–(C=O) at ∼534.3 eV). These are mainly ejected from SEI, and are lower for modified electrolytes, indicating thinner SEI film. Another point to note here is that the SEI compounds such as alcohols (C–O), carbonyls (C=O), esters (O–C=O), and carbonate (CO_3_) groups also contribute to the C 1s spectrum. Therefore, a detailed picture of the C 1s spectrum is presented to separate out the signals collected from graphite active material only and the above-mentioned SEI components. In Figure 8b, the combined percentage concentration of SEI components such as C–O, C=O, O=C–O, CO_3_ bonds are significantly lower for E-0.001M electrolyte (at 2C rate), suggesting thinner SEI film on the graphite surface. A similar trend is also followed at the 3C rate, shown in Figure 8c. The higher percentage concentration of sp^3^ C (and lower concentration of signals associate with SEI components, in Figure 8c) supports the notion of a thinner SEI in E-0.001M compared with all the other electrolytes. The presence of lithium acetylide denotes that Li dendrite formation is not completely inhibited at the 3C rate, previously shown in Figure 6f.

**Figure 9:**
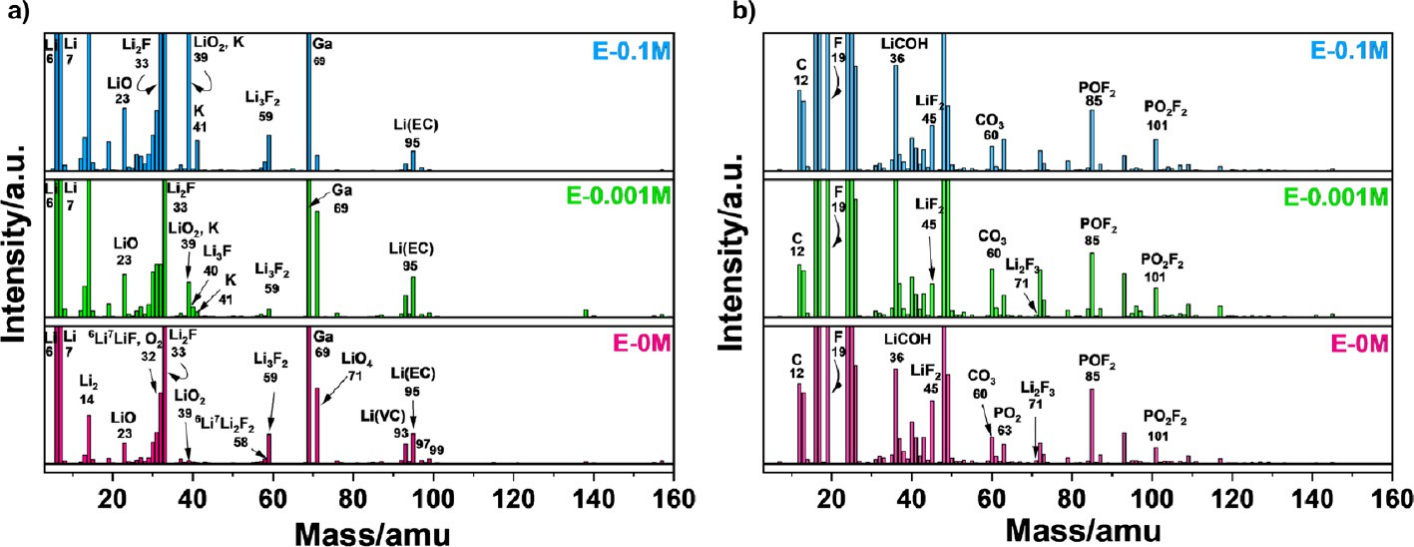
SIMS (a) positive ion and (b) negative ion mode mass spectra of graphite anode surface using E-0M, E-0.001M, and E-0.1M electrolyte.

It has been previously reported that the grain boundaries, cracks, pores, and the heterogeneous interfaces present in the SEI film are responsible for creating pathways for electron leakage due to their lower energy barriers.^62^ The electrons pass through the SEI layer via above-mentioned defect sites, resulting in electrolyte decomposition and Li^+^ to Li metal reduction. The dense inorganic SEI components (located closer to the electrode), when present in sufficient quantity, act as a blockage to the electron leakage pathways, restricting in metallic Li deposition and electrolyte decomposition. Among all, LiF is the prominent inorganic SEI component, which provides better surface passivation and increases the stability and robustness of SEI films. The insulating nature of LiF compound (∼10^–13^ to 10^–14^ S/cm)^63^ provides high resistance to electron transport (through SEI layer), which could have contributed to Li^+^ to Li^0^ reduction. Furthermore, low solubility, low Li^+^ diffusion barrier, and large Li^+^ diffusion coefficient across the LiF surface suggest a faster Li^+^ diffusion rate, implying excellent SEI stability.^25,63,64^ In addition, the LiF-rich layer has reportedly improved the morphology of the anode by homogenizing Li^+^ flux during Li dendrite formation and growth process.^65^ Therefore, the nanocrystals of LiF facilitate the uniform transportation of Li^+^ thereby restricting Li dendrite growth.^15,64,66^ Hence, the F 1s spectrum is further explored to investigate the metal fluoride present in the SEI film. Figure 8d shows that the percentage concentration of metal fluoride is highest in E-0.001M modified electrolyte and lowest in E-0M commercial electrolyte. This specifies that E-0.001M (with higher metal fluoride concentration) can effectively block the developed electron leakage pathways in the SEI film, thus blocking the electrons that could contribute to the reduction process of Li^+^ to Li^0^ (metallic Li) and its growth thereafter. Therefore, Li dendrites are not observed in E-0.001M optimized electrolyte (Figures 3d, 6e, and S5e), whereas commercial E-0M electrolyte experiences Li dendrites at the edge of graphite particles (Figures 3d and 4d). A similar observation is observed at a 3C charging rate, where the percentage concentration of metal fluoride in E-0.001M electrolyte is highest among all the electrolytes, shown in Figure 8e. Although Li dendrite formation is not completely inhibited at the 3C rate, its growth is clearly restricted (Figure 6f) compared to the E-0M commercial electrolyte (Figure 4e). Moreover, 0.001 M KPF_6_ is shown to be the optimal concentration for controlling the growth of Li dendrites on graphite. It should be noted that “metal fluoride” is mentioned instead of LiF in Figure 8d,e, as fluoride could be associated with Li only or a combination of Li and K. To investigate this, SIMS was carried out and the mass spectra for E-0M, E-0.001M, and E-0.1M electrolytes are presented in Figure 9.

Figure 9 provides the information regarding the ion clusters of organic and inorganic compounds of the SEI layer present on the graphite surface. The collected ion fragments are listed in Table S3 in Supporting Information. Li isotopes, i.e., ^6^Li^+^, ^7^Li^+^, and ^19^F^–^ are the principal peaks in positive and negative ion modes, respectively. The positive ion fragments, e.g., ^6^Li^7^LiF^+^ (32 amu), ^7^Li_2_F^+^ (33 amu), ^7^Li_3_F^+^ (40 amu), ^7^Li_2_F_2_^+^ (52 amu), ^6^Li^7^Li_2_F^+^ (58 amu)^+^, ^7^Li_3_F_2_^+^ (59 amu) correspond to lithium fluoride (LiF).^67−72^ Similarly, the negative ion fragments e.g., ^6^LiF_2_^–^ (44 amu), ^7^LiF_2_^–^ (45 amu), ^7^Li_2_F_3_^–^ (71 amu) reveals the existence of LiF in the SEI film. These positive and negative ion fragments are observed not only in E-0M commercial electrolyte but also in E-0.001M and E-0.1M, suggesting LiF presence in the SEI film in all of the electrolytes. It should be noted that the intensities of peaks present at 39 and 41 amu are significantly increased in E-0.001M and E-0.1M (compared to E-0M), indicating the presence of ^39^K^+^ and ^41^K^+^ (potassium additive incorporation into electrolyte). In order for potassium fluoride (KF) to be present in SEI, the ion species such as ^39^K_2_F^+^ (97 amu), ^39^K^41^KF^+^ (99 amu), ^39^K_3_F_2_^+^ (155 amu), ^39^K_2_^41^KF^+^ (157 amu), and ^39^KF_2_^–^ (77 amu), ^41^KF_2_^–^ (79 amu), ^39^K_2_F_3_^–^ (135 amu), ^39^K^41^KF_3_^–^ (137 amu) are anticipated in positive and negative ion modes, respectively.^36,73,74^ There are two small peaks recorded at 97 and 99 amu in positive ion mode for E-0M electrolyte; however, the intensities remain the same in the cases of E-0.001M and E-0.1M. A similar observation is measured at 155 and 157 amu in negative ion mode. In addition to this, the relative abundance ratio of potassium isotopes, i.e., ^39^K^+^/^41^K^+^ = 13.8,^75^ does not match with the intensity ratios of the peaks present at 99 and 97 amu in positive ion mode and at 155 and 157 amu in negative ion mode. This confirms that the increase in metal fluoride concentration is due to increment in LiF content only. KF is not present in the SEI film on the surface of the graphite anode. The higher solubility of KF (compared to LiF) could be the reason behind this behavior.^76^ For depth analysis, SIMS was carried out at the same location, presented in Figure S7. The ratio of the peaks in both positive and negative ion modes, e.g., at 97 amu/99 amu and at 155 amu/157 amu, respectively, is inspected again and found out to be unmatched with ^39^K^+^/^41^K^+^, confirming no KF present in the SEI layer. Therefore, the peaks at 97 amu, 99 amu, 79 amu, and 137 amu correspond to C_5_H_5_O_2_^+^, C_5_H_7_O_2_^+^, PO_3_^–^, and C_3_H_6_PO_4_^–^ ion fragments in positive and negative ion modes. In Figure S8, it is seen that potassium is deposited at certain preferential sites such as defects, particle edges, and grain boundaries, similar to Li^+^. Therefore, in E-0.001M optimized electrolyte, K^+^ deposits at the defect sites due to its faster diffusion rate (in the electrolyte) and lower desolvation energy compared to Li^+^ (because of smaller Stokes radius of solvated K^+^ ion).^36,37^ Additionally, K^+^ occupying the defect sites prevents the incoming Li^+^ from participating in Li dendritic formation and growth, thereby reducing the probability of Li^+^ reduction to Li^0^. Furthermore, thin LiF-rich SEI layer blocks the electron leakage pathways for possible Li^+^ reduction to metallic Li. K^+^ deposition on graphite defect sites along with highest LiF content in SEI film blocks the respective incoming Li^+^ and the electrons in E-0.001M, thereby suppressing the dendrite growth. LiF content is decreased in all other concentrations of additive as K^+^ is consumed (reduced to K metal) and henceforth cannot effectively block all the defect sites. This leads to the formation and growth of Li metal dendrites at these defect sites. Moreover, Li^+^ is reduced to Li metal instead of reacting with F for LiF formation and therefore is ineffective in controlling dendrite growth with increased additive concentration.

### 3.3 AC Impedance Characterization

**Figure 10:**
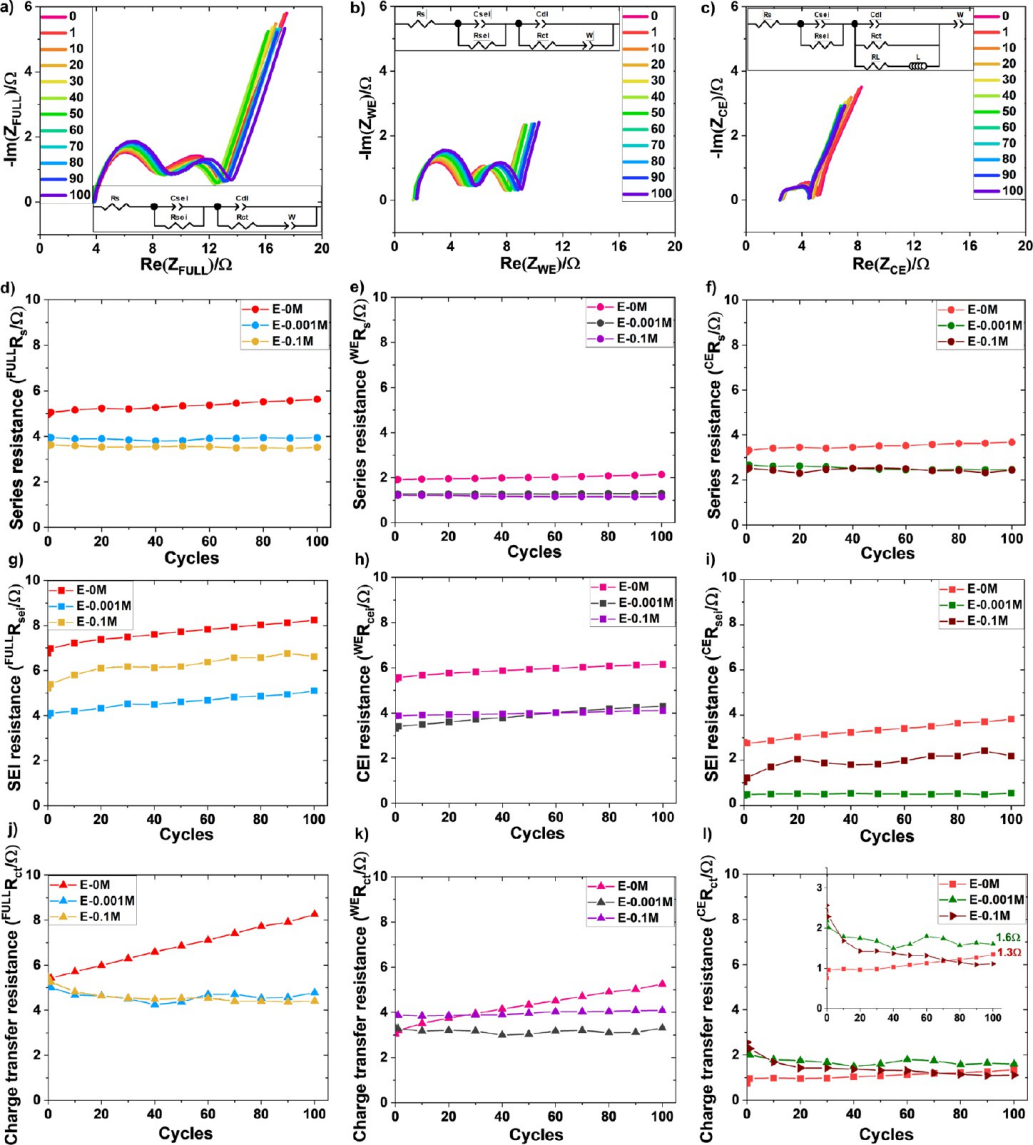
EIS Nyquist plot of three electrode EL-cell (graphite|Li∥NMC 622|Li) showing (a) full cell (graphite|NMC 622) spectra, (b) cathode half cell (NMC 622|Li), (c) anode half cell (graphite|Li) spectra of E-0.001M electrolyte at 2C rate with equivalent circuit modeling (ECM). Comparison of (d, e, f) *R*_s_, (g, h, i) *R*_sei_, and (j, k, l) *R*_ct_ in of electrolytes (E-0M, E-0.001M, and E-0.1M) upon cycles.

Figure 10 presents Nyquist plots using three-electrode EL-cells incorporating three different electrolytes, e.g., E-0.1M, and E-0.001M optimized electrolyte and E-0M commercial electrolyte for reference at 2C rate. The resistance results obtained from equivalent circuit modeling (ECM) are plotted with respect to cycle number, also shown in Figure 10. The ohmic resistance or series resistance (*R*_s_) is increased over the cycles for all the electrolytes for both full cell and half cells. The lower series resistances (*R*_s_) for additive based electrolytes indicate their increased ionic conductivity compared to E-0M commercial electrolyte. SEI resistances (*R*_sei_) presented in Figure 10g–i show that E-0.001M has lower SEI and CEI (cathode electrolyte interphase) resistances compared to other electrolytes. This is because thinner LiF-rich SEI (Figure 8) in E-0.001M electrolyte facilitates lower resistance to Li^+^ mass transport. However, in the case of E-0M commercial electrolyte, Li dendrites promote more electrolyte decomposition (Figure 4d) and hence thickens the SEI layer, resulting in higher resistance to Li^+^ transport. Figure 10j–l shows the charge-transfer resistance (*R*_ct_) for all the electrolytes. It is noticed that the *R*_ct_ value is increased with successive cycles for E-0M due to Li dendrite formation leading to slower electrochemical kinetics of the cell. However, the trend is different for modified electrolytes, where the *R*_ct_ value remains almost stable with progressive cycles for full cell and cathode half cell, shown in Figure 10j,k. This steady *R*_ct_ indicates the kinetic stability of additive based electrolytes. In the case of anode half cell (graphite|Li) in Figure 10l), *R*_ct_ of additive-based electrolytes (E-0.001M and E-0.1M) is slightly higher (compared to E-0M) for the initial few cycles and decreases afterward to remain stable upon further cycling. It is observed that the *R*_ct_ value is in increasing trend in E-0M electrolyte (zoomed in Figure 10l), meaning that the *R*_ct_ value is expected to increase even after the 100th cycle (as cycles progresses) with the growth of Li dendrite. In additive-based electrolytes, the SEI formation through electrolyte decomposition could contribute to its higher *R*_ct_ for the initial few cycles. Once the SEI film becomes thermodynamically stabilized, the resistance to charge-transfer (*R*_ct_) is decreased subsequently implying electrochemical kinetic stability of the cell. Moreover, the difference in *R*_ct_ values between E-0M (1.3 Ω) and E-0.001M (1.6 Ω), E-0.1M (1.1 Ω) is negligible. Finally, the difference in *R*_ct_ values is distinctively visible in a full cell, i.e., Figure 10j, indicating higher *R*_ct_ value in commercial E-0M electrolyte compared to optimum E-0.001M. This suggests the positive impact of KPF_6_ additive in controlling Li dendrite growth on graphite anode.

## 4 Conclusion

K additives, in appropriate concentrations, can play a crucial role toward the formation of Li dendrites and their growth. The incorporation of a KPF_6_ electrolyte additive is comprehensively investigated by systematically varying the concentrations in full and three electrode cells. Higher concentrations such as 0.15 and 0.2 M KPF_6_ are detrimental to the cells’ performance as K^+^ gets reduced and forms its own dendrites. These potassium dendrites cover the surface of the graphite anode, which impedes the transport of Li^+^ through the graphite layers. 0.001 M KPF_6_ concentration was concluded to be the optimized concentration by enabling a thin LiF-rich SEI film, facilitating faster Li^+^ transport. Higher LiF content blocks the potential electron leakage pathways for Li^+^ reduction to Li^0^ metal. Additionally, the faster diffusion rate of K^+^ in the electrolyte and its lower desolvation energy block the defect sites for favorable Li dendrite nucleation. Both of these processes act simultaneously to generate a dendritic-free faster-charging graphite anode, which can critically influence the development of improved high-rate cell chemistries. With further consideration and development given to optimizing the microstructure formulation of the anodes, it is expected that overall performance will also become much more improved relating to capacity retention also.

## Supporting Information Available

Information about electrodes, figure of EL-cell components, electrochemical cycling data, parasitic reaction rate, SEM images of cycled graphite anodes with different electrolytes, Nernst equation derivation for potassium deposition, and additional XPS, SIMS, XRF results (PDF)

## References

[ref1] Reddy M. V.; Mauger A.; Julien C. M.; Paolella A.; Zaghib K. Brief History of Early Lithium-Battery Development. Materials 2020, 13 (8), 1884 10.3390/ma13081884. PMC7215417 32316390

[ref2] Daniel C.; Mohanty D.; Li J.; Wood D. L. Cathode Materials Review. AIP Conf. Proc. 2014, 1597, 26–43. 10.1063/1.4878478.

[ref3] Shen X.; Zhang X.-Q.; Ding F.; Huang J.-Q.; Xu R.; Chen X.; Yan C.; Su F.-Y.; Chen C.-M.; Liu X.; Zhang Q. Advanced Electrode Materials in Lithium Batteries: Retrospect and Prospect. Energy Material Advances 2021, 2021, 1–15. 10.34133/2021/1205324.

[ref4] An S. J.; Li J.; Daniel C.; Mohanty D.; Nagpure S.; Wood D. L. The State of Understanding of the Lithium-Ion-Battery Graphite Solid Electrolyte Interphase (SEI) and Its Relationship to Formation Cycling. Carbon. 2016, 105, 52–76. 10.1016/j.carbon.2016.04.008.

[ref5] Tomaszewska A.; Chu Z.; Feng X.; O’Kane S.; Liu X.; Chen J.; Ji C.; Endler E.; Li R.; Liu L.; Li Y.; Zheng S.; Vetterlein S.; Gao M.; Du J.; Parkes M.; Ouyang M.; Marinescu M.; Offer G.; Wu B. Lithium-Ion Battery Fast Charging: A Review. eTransportation 2019, 1, 100011 10.1016/j.etran.2019.100011.

[ref6] Tallman K. R.; Zhang B.; Wang L.; Yan S.; Thompson K.; Tong X.; Thieme J.; Kiss A.; Marschilok A. C.; Takeuchi K. J.; Bock D. C.; Takeuchi E. S. Anode Overpotential Control via Interfacial Modification: Inhibition of Lithium Plating on Graphite Anodes. ACS Appl. Mater. Interfaces 2019, 11 (50), 46864–46874. 10.1021/acsami.9b16794. 31755690

[ref7] Legrand N.; Knosp B.; Desprez P.; Lapicque F.; Raël S. Physical Characterization of the Charging Process of a Li-Ion Battery and Prediction of Li Plating by Electrochemical Modelling. J. Power Sources 2014, 245, 208–216. 10.1016/j.jpowsour.2013.06.130.

[ref8] Kong L.; Xing Y.; Pecht M. G. In-Situ Observations of Lithium Dendrite Growth. IEEE Access 2018, 6, 8387–8393. 10.1109/ACCESS.2018.2805281.

[ref9] Wu B.; Wang S.; Lochala J.; Desrochers D.; Liu B.; Zhang W.; Yang J.; Xiao J. The Role of the Solid Electrolyte Interphase Layer in Preventing Li Dendrite Growth in Solid-State Batteries. Energy Environ. Sci. 2018, 11 (7), 1803–1810. 10.1039/C8EE00540K.

[ref10] Cao D.; Sun X.; Li Q.; Natan A.; Xiang P.; Zhu H. Lithium Dendrite in All-Solid-State Batteries: Growth Mechanisms, Suppression Strategies, and Characterizations. Matter. 2020, 3, 57–94. 10.1016/j.matt.2020.03.015.

[ref11] Barai P.; Higa K.; Srinivasan V. Lithium Dendrite Growth Mechanisms in Polymer Electrolytes and Prevention Strategies. Phys. Chem. Chem. Phys. 2017, 19 (31), 20493–20505. 10.1039/C7CP03304D. 28726884

[ref12] Chu F.; Hu J.; Tian J.; Zhou X.; Li Z.; Li C. In Situ Plating of Porous Mg Network Layer to Reinforce Anode Dendrite Suppression in Li-Metal Batteries. ACS Appl. Mater. Interfaces 2018, 10 (15), 12678–12689. 10.1021/acsami.8b00989. 29569892

[ref13] Meng J.; Lei M.; Lai C.; Wu Q.; Liu Y.; Li C. Lithium Ion Repulsion-Enrichment Synergism Induced by Core-Shell Ionic Complexes to Enable High-Loading Lithium Metal Batteries. Angewandte Chemie - International Edition 2021, 60 (43), 23256–23266. 10.1002/anie.202108143. 34405939

[ref14] Hu J.; Chen K.; Li C. Nanostructured Li-Rich Fluoride Coated by Ionic Liquid as High Ion-Conductivity Solid Electrolyte Additive to Suppress Dendrite Growth at Li Metal Anode. ACS Appl. Mater. Interfaces 2018, 10 (40), 34322–34331. 10.1021/acsami.8b12579. 30207450

[ref15] Chandrasiri K. W. D. K.; Nguyen C. C.; Zhang Y.; Parimalam B. S.; Lucht B. L. Systematic Investigation of Alkali Metal Ions as Additives for Graphite Anode in Propylene Carbonate Based Electrolytes. Electrochim. Acta 2017, 250, 285–291. 10.1016/j.electacta.2017.08.065.

[ref16] Xiang H.; Mei D.; Yan P.; Bhattacharya P.; Burton S. D.; Von Wald Cresce A.; Cao R.; Engelhard M. H.; Bowden M. E.; Zhu Z.; Polzin B. J.; Wang C. M.; Xu K.; Zhang J. G.; Xu W. The Role of Cesium Cation in Controlling Interphasial Chemistry on Graphite Anode in Propylene Carbonate-Rich Electrolytes. ACS Appl. Mater. Interfaces 2015, 7 (37), 20687–20695. 10.1021/acsami.5b05552. 26369297

[ref17] Ding M. S.; Li Q.; Li X.; Xu W.; Xu K. Effects of Solvent Composition on Liquid Range, Glass Transition, and Conductivity of Electrolytes of a (Li, Cs)PF6 Salt in EC-PC-EMC Solvents. J. Phys. Chem. C 2017, 121 (21), 11178–11183. 10.1021/acs.jpcc.7b03306.

[ref18] Ding F.; Xu W.; Graff G. L.; Zhang J.; Sushko M. L.; Chen X.; Shao Y.; Engelhard M. H.; Nie Z.; Xiao J.; Liu X.; Sushko P. V.; Liu J.; Zhang J. G. Dendrite-Free Lithium Deposition via Self-Healing Electrostatic Shield Mechanism. J. Am. Chem. Soc. 2013, 135 (11), 4450–4456. 10.1021/ja312241y. 23448508

[ref19] Kubota K.; Matsumoto H. Cation Mixtures of Alkali Metal (Fluorosulfonyl)(Trifluoromethylsulfonyl)Amide as Electrolytes for Lithium Secondary Battery. J. Electrochem. Soc. 2014, 161 (6), A902–A907. 10.1149/2.026406jes.

[ref20] Xiao L.; Chen X.; Cao R.; Qian J.; Xiang H.; Zheng J.; Zhang J. G.; Xu W. Enhanced Performance of Li|LiFePO4 Cells Using CsPF6 as an Electrolyte Additive. J. Power Sources 2015, 293, 1062–1067. 10.1016/j.jpowsour.2015.06.044.

[ref21] Zheng J.; Yan P.; Cao R.; Xiang H.; Engelhard M. H.; Polzin B. J.; Wang C.; Zhang J. G.; Xu W. Effects of Propylene Carbonate Content in CsPF6-Containing Electrolytes on the Enhanced Performances of Graphite Electrode for Lithium-Ion Batteries. ACS Appl. Mater. Interfaces 2016, 8 (8), 5715–5722. 10.1021/acsami.5b12517. 26862677

[ref22] Komaba S.; Watanabe M.; Groult H.; Kumagai N.; Okahara K. Impact of Sodium Salt Coating on a Graphite Negative Electrode for Lithium-Ion Batteries. Electrochem. Solid-State Lett. 2006, 9 (3), A130 10.1149/1.2161453.

[ref23] Goodman J. K. S.; Kohl P. A. Effect of Alkali and Alkaline Earth Metal Salts on Suppression of Lithium Dendrites. J. Electrochem. Soc. 2014, 161 (9), D418–D424. 10.1149/2.0301409jes.

[ref24] Komaba S.; Itabashi T.; Watanabe M.; Groult H.; Kumagai N. Electrochemistry of Graphite in Li and Na Salt Codissolving Electrolyte for Rechargeable Batteries. J. Electrochem. Soc. 2007, 154 (4), A322 10.1149/1.2472552.

[ref25] Wu F.; Yuan Y. X.; Cheng X. B.; Bai Y.; Li Y.; Wu C.; Zhang Q. Perspectives for Restraining Harsh Lithium Dendrite Growth: Towards Robust Lithium Metal Anodes. Energy Storage Materials. 2018, 15, 148–170. 10.1016/j.ensm.2018.03.024.

[ref26] Wood S. M.; Pham C. H.; Rodriguez R.; Nathan S. S.; Dolocan A. D.; Celio H.; De Souza J. P.; Klavetter K. C.; Heller A.; Mullins C. B. K+ Reduces Lithium Dendrite Growth by Forming a Thin, Less-Resistive Solid Electrolyte Interphase. ACS Energy Letters 2016, 1 (2), 414–419. 10.1021/acsenergylett.6b00259.

[ref27] Zhang K.; Wu F.; Zhang K.; Weng S.; Wang X.; Gao M.; Sun Y.; Cao D.; Bai Y.; Xu H.; Wang X.; Wu C. Chlorinated Dual-Protective Layers as Interfacial Stabilizer for Dendrite-Free Lithium Metal Anode. Energy Storage Materials 2021, 41, 485–494. 10.1016/j.ensm.2021.06.023.

[ref28] Zhuang Q. C.; Li J.; Tian L. L. Potassium Carbonate as Film Forming Electrolyte Additive for Lithium-Ion Batteries. J. Power Sources 2013, 222, 177–183. 10.1016/j.jpowsour.2012.08.050.

[ref29] Zheng H.; Fu Y.; Zhang H.; Abe T.; Ogumi Z. Potassium Salts: Electrolyte Additives for Enhancing Electrochemical Performances of Natural Graphite Anodes. Electrochem. Solid-State Lett. 2006, 9 (3), A115 10.1149/1.2161447.

[ref30] Tossici R.; Berrettoni M.; Nalimova V.; Marassi R.; Scrosati B. A High-Rate Carbon Electrode for Rechargeable Lithium-Ion Batteries. J. Electrochem. Soc. 1996, 143 (3), L64–L67. 10.1149/1.1836534.

[ref31] Xia W.; Peng Q.; Zhang Z.; Yang L.; Fu Y.; Wang X. Effects of KPF6 on the Electrochemical Performance of Natural Graphite/Li. Ionics 2015, 21 (12), 3177–3184. 10.1007/s11581-015-1513-0.

[ref32] Komaba S.; Watanabe M.; Groult H.; Kumagai N. Alkali Carbonate-Coated Graphite Electrode for Lithium-Ion Batteries. Carbon 2008, 46 (9), 1184–1193. 10.1016/j.carbon.2008.04.021.

[ref33] Komaba S.; Itabashi T.; Kimura T.; Groult H.; Kumagai N. Opposite Influences of K+ versus Na+ Ions as Electrolyte Additives on Graphite Electrode Performance. J. Power Sources 2005, 146, 166–170. 10.1016/j.jpowsour.2005.03.121.

[ref34] Pathan T. S.; Rashid M.; Walker M.; Widanage W. D.; Kendrick E. Active Formation of Li-Ion Batteries and Its Effect on Cycle Life. J. Phys.: Energy 2019, 1 (4), 044003 10.1088/2515-7655/ab2e92.

[ref35] Jung R.; Metzger M.; Maglia F.; Stinner C.; Gasteiger H. A. Oxygen Release and Its Effect on the Cycling Stability of LiNi x Mn y Co z O 2 (NMC) Cathode Materials for Li-Ion Batteries. J. Electrochem. Soc. 2017, 164 (7), A1361–A1377. 10.1149/2.0021707jes.

[ref36] Hosaka T.; Kubota K.; Hameed A. S.; Komaba S. Research Development on K-Ion Batteries. Chem. Rev. 2020, 120, 6358–6466. 10.1021/acs.chemrev.9b00463. 31939297

[ref37] Okoshi M.; Yamada Y.; Komaba S.; Yamada A.; Nakai H. Theoretical Analysis of Interactions between Potassium Ions and Organic Electrolyte Solvents: A Comparison with Lithium, Sodium, and Magnesium Ions. J. Electrochem. Soc. 2017, 164 (2), A54–A60. 10.1149/2.0211702jes.

[ref38] Dahn J. R. Phase Diagram of LixC6. Phys. Rev. B 1991, 44 (17), 9170–9177. 10.1103/PhysRevB.44.9170. 9998896

[ref39] Asenbauer J.; Eisenmann T.; Kuenzel M.; Kazzazi A.; Chen Z.; Bresser D. The Success Story of Graphite as a Lithium-Ion Anode Material-Fundamentals, Remaining Challenges, and Recent Developments Including Silicon (Oxide) Composites. Sustainable Energy and Fuels. 2020, 4, 5387–5416. 10.1039/D0SE00175A.

[ref40] Heß M.; Novák P. Shrinking Annuli Mechanism and Stage-Dependent Rate Capability of Thin-Layer Graphite Electrodes for Lithium-Ion Batteries. Electrochim. Acta 2013, 106, 149–158. 10.1016/j.electacta.2013.05.056.

[ref41] Guo Z.; Zhu J.; Feng J.; Du S. Direct in Situ Observation and Explanation of Lithium Dendrite of Commercial Graphite Electrodes. RSC Adv. 2015, 5 (85), 69514–69521. 10.1039/C5RA13289D.

[ref42] Bai P.; Li J.; Brushett F. R.; Bazant M. Z. Transition of Lithium Growth Mechanisms in Liquid Electrolytes. Energy Environ. Sci. 2016, 9 (10), 3221–3229. 10.1039/C6EE01674J.

[ref43] Kumar A.; Arruda T. M.; Tselev A.; Ivanov I. N.; Lawton J. S.; Zawodzinski T. A.; Butyaev O.; Zayats S.; Jesse S.; Kalinin S. V. Nanometer-Scale Mapping of Irreversible Electrochemical Nucleation Processes on Solid Li-Ion Electrolytes. Sci. Rep. 2013, 3, 1621 10.1038/srep01621. 23563856 PMC3619134

[ref44] Bhattacharya S.; Alpas A. T. Micromechanisms of Solid Electrolyte Interphase Formation on Electrochemically Cycled Graphite Electrodes in Lithium-Ion Cells. Carbon 2012, 50 (15), 5359–5371. 10.1016/j.carbon.2012.07.009.

[ref45] Cabo-Fernandez L.; Neale A. R.; Braga F.; Sazanovich I. V.; Kostecki R.; Hardwick L. J. Kerr Gated Raman Spectroscopy of LiPF6 Salt and LiPF6-Based Organic Carbonate Electrolyte for Li-Ion Batteries. Phys. Chem. Chem. Phys. 2019, 21 (43), 23833–23842. 10.1039/C9CP02430A. 31538641

[ref46] Cresce A. V.; Russell S. M.; Borodin O.; Allen J. A.; Schroeder M. A.; Dai M.; Peng J.; Gobet M. P.; Greenbaum S. G.; Rogers R. E.; Xu K. Solvation Behavior of Carbonate-Based Electrolytes in Sodium Ion Batteries. Phys. Chem. Chem. Phys. 2017, 19 (1), 574–586. 10.1039/C6CP07215A. 27918030

[ref47] Gibbs J. W.; Mohan K. A.; Gulsoy E. B.; Shahani A. J.; Xiao X.; Bouman C. A.; De Graef M.; Voorhees P. W. The Three-Dimensional Morphology of Growing Dendrites. Sci. Rep. 2015, 5, 11824 10.1038/srep11824. 26139473 PMC4490335

[ref48] Kim Y. J.; Kwon S. H.; Noh H.; Yuk S.; Lee H.; Jin H. s.; Lee J.; Zhang J. G.; Lee S. G.; Guim H.; Kim H. T. Facet Selectivity of Cu Current Collector for Li Electrodeposition. Energy Storage Mater. 2019, 19, 154–162. 10.1016/j.ensm.2019.02.011.

[ref49] Swetha P.; Feng S. P. High-Index Facet Defined Shape-Controlled Electrochemical Synthesis of Nanocrystals: A Mini Review. Electrochem. Commun. 2018, 94, 64–69. 10.1016/j.elecom.2018.08.007.

[ref50] Díaz J.; Paolicelli G.; Ferrer S.; Comin F. Separation of the and Components in the C1s Photoemission Spectra of Amorphous Carbon Films. Physical Review B - Condensed Matter and Materials Physics 1996, 54 (11), 8064–8069. 10.1103/PhysRevB.54.8064. 9984485

[ref51] Oswald S.; Thoss F.; Zier M.; Hoffmann M.; Jaumann T.; Herklotz M.; Nikolowski K.; Scheiba F.; Kohl M.; Giebeler L.; Mikhailova D.; Ehrenberg H. Binding Energy Referencing for XPS in Alkali Metal-Based Battery Materials Research (II): Application to Complex Composite Electrodes. Batteries 2018, 4 (3), 36 10.3390/batteries4030036.

[ref52] Greczynski G.; Hultman L. X-Ray Photoelectron Spectroscopy: Towards Reliable Binding Energy Referencing. Prog. Mater. Sci. 2020, 107, 100591 10.1016/j.pmatsci.2019.100591.

[ref53] Biesinger M. C. X-ray Photoelectron Sepctroscopy (XPS) Reference Pages. http://www.xpsfitting.com/search/label/Phosphorus.

[ref54] Tan C. C.; Walker M.; Remy G.; Kourra N.; Maddar F.; Dixon S.; Williams M.; Loveridge M. J. Ageing Analysis and Asymmetric Stress Considerations for Small Format Cylindrical Cells for Wearable Electronic Devices. J. Power Sources 2020, 472, 228626 10.1016/j.jpowsour.2020.228626.

[ref55] Rubio Lopez I.; Lain M. J.; Kendrick E. Optimisation of Formation and Conditioning Protocols for Lithium-Ion Electric Vehicle Batteries. Batteries & Supercaps 2020, 3 (9), 900–909. 10.1002/batt.202000048.

[ref56] Fonseca Rodrigues M. T.; Maroni V. A.; Gosztola D. J.; Yao K. P. C.; Kalaga K.; Shkrob I. A.; Abraham D. P. Lithium Acetylide: A Spectroscopic Marker for Lithium Deposition during Fast Charging of Li-Ion Cells. ACS Applied Energy Materials 2019, 2 (1), 873–881. 10.1021/acsaem.8b01975.

[ref57] Su X.; Dogan F.; Ilavsky J.; Maroni V. A.; Gosztola D. J.; Lu W. Mechanisms for Lithium Nucleation and Dendrite Growth in Selected Carbon Allotropes. Chem. Mater. 2017, 29 (15), 6205–6213. 10.1021/acs.chemmater.7b00072.

[ref58] Schmitz R.; Müller R.; Krüger S.; Schmitz R. W.; Nowak S.; Passerini S.; Winter M.; Schreiner C. Investigation of Lithium Carbide Contamination in Battery Grade Lithium Metal. J. Power Sources 2012, 217, 98–101. 10.1016/j.jpowsour.2012.05.038.

[ref59] Tang S.; Gu Y.; Yi J.; Zeng Z.; Ding S. Y.; Yan J. W.; Wu D. Y.; Ren B.; Tian Z. Q.; Mao B. W. An Electrochemical Surface-Enhanced Raman Spectroscopic Study on Nanorod-Structured Lithium Prepared by Electrodeposition. J. Raman Spectrosc. 2016, 47 (9), 1017–1023. 10.1002/jrs.4970.

[ref60] Woo J.-J.; Maroni V. A.; Liu G.; Vaughey J. T.; Gosztola D. J.; Amine K.; Zhang Z. Symmetrical Impedance Study on Inactivation Induced Degradation of Lithium Electrodes for Batteries Beyond Lithium-Ion. J. Electrochem. Soc. 2014, 161 (5), A827–A830. 10.1149/2.089405jes.

[ref61] Biesinger M. C. XPS Detection Limits:X-ray Photoelectron Spectroscopy (XPS) Reference Pages. http://www.xpsfitting.com/2017/05/xps-detection-limits.html.

[ref62] Feng M.; Pan J.; Qi Y. Impact of Electronic Properties of Grain Boundaries on the Solid Electrolyte Interphases (SEIs) in Li-Ion Batteries. J. Phys. Chem. C 2021, 125 (29), 15821–15829. 10.1021/acs.jpcc.1c03186.

[ref63] He M.; Guo R.; Hobold G. M.; Gao H.; Gallant B. M. The Intrinsic Behavior of Lithium Fluoride in Solid Electrolyte Interphases on Lithium. Proc. Natl. Acad. Sci. U.S.A. 2020, 117 (1), 73–79. 10.1073/pnas.1911017116. 31848237 PMC6955333

[ref64] Zheng J.; Ju Z.; Zhang B.; Nai J.; Liu T.; Liu Y.; Xie Q.; Zhang W.; Wang Y.; Tao X. Lithium Ion Diffusion Mechanism on the Inorganic Components of the Solid-Electrolyte Interphase. Journal of Materials Chemistry A 2021, 9 (16), 10251–10259. 10.1039/D0TA11444H.

[ref65] Yuan Y.; Wu F.; Bai Y.; Li Y.; Chen G.; Wang Z.; Wu C. Regulating Li Deposition by Constructing LiF-Rich Host for Dendrite-Free Lithium Metal Anode. Energy Storage Materials 2019, 16, 411–418. 10.1016/j.ensm.2018.06.022.

[ref66] Li C.; Gu L.; Maier J. Enhancement of the Li Conductivity in LiF by Introducing Glass/Crystal Interfaces. Adv. Funct. Mater. 2012, 22 (6), 1145–1149. 10.1002/adfm.201101798.

[ref67] Li W.; Kim U. H.; Dolocan A.; Sun Y. K.; Manthiram A. Formation and Inhibition of Metallic Lithium Microstructures in Lithium Batteries Driven by Chemical Crossover. ACS Nano 2017, 11 (6), 5853–5863. 10.1021/acsnano.7b01494. 28502161

[ref68] Zhang Y.; Su M.; Yu X.; Zhou Y.; Wang J.; Cao R.; Xu W.; Wang C.; Baer D. R.; Borodin O.; Xu K.; Wang Y.; Wang X. L.; Xu Z.; Wang F.; Zhu Z. Investigation of Ion-Solvent Interactions in Nonaqueous Electrolytes Using in Situ Liquid SIMS. Anal. Chem. 2018, 90 (5), 3341–3348. 10.1021/acs.analchem.7b04921. 29405699

[ref69] Ota H.; Akai T.; Namita H.; Yamaguchi S.; Nomura M. XAFS and TOF-SIMS Analysis of SEI Layers on Electrodes. J. Power Sources 2003, 119–121, 567–571. 10.1016/S0378-7753(03)00291-X.

[ref70] Lu J.; Hua X.; Long Y. T. Recent Advances in Real-Time and in Situ Analysis of an Electrode-Electrolyte Interface by Mass Spectrometry. Analyst. 2017, 142, 691–699. 10.1039/C6AN02757A. 28180217

[ref71] Wheatcroft L.; Klingner N.; Heller R.; Hlawacek G.; Özkaya D.; Cookson J.; Inkson B. J. Visualization and Chemical Characterization of the Cathode Electrolyte Interphase Using He-Ion Microscopy and in Situ Time-of-Flight Secondary Ion Mass Spectrometry. ACS Applied Energy Materials 2020, 3 (9), 8822–8832. 10.1021/acsaem.0c01333. 33015588 PMC7525808

[ref72] Zhou Y.; Su M.; Yu X.; Zhang Y.; Wang J. G.; Ren X.; Cao R.; Xu W.; Baer D. R.; Du Y.; Borodin O.; Wang Y.; Wang X. L.; Xu K.; Xu Z.; Wang C.; Zhu Z. Real-Time Mass Spectrometric Characterization of the Solid-Electrolyte Interphase of a Lithium-Ion Battery. Nat. Nanotechnol. 2020, 15 (3), 224–230. 10.1038/s41565-019-0618-4. 31988500

[ref73] Wang H.; Zhai D.; Kang F. Solid Electrolyte Interphase (SEI) in Potassium Ion Batteries. Energy and Environmental Science. 2020, 13, 4583–4608. 10.1039/D0EE01638A.

[ref74] Zhang Y.; Zeng W.; Huang L.; Liu W.; Jia E.; Zhao Y.; Wang F.; Zhu Z. In Situ Liquid Secondary Ion Mass Spectrometry: A Surprisingly Soft Ionization Process for Investigation of Halide Ion Hydration. Anal. Chem. 2019, 91 (11), 7039–7046. 10.1021/acs.analchem.8b05804. 30950268

[ref75] Garner E. L.; Murphy T. J.; Gramlich J. W.; Paulsen P. J.; Barnes I. L. Absolute Isotopic Abundance Ratios and the Atomic Weight of a Reference Sample of Potassium. J. Res. Natl. Bur. Stand., Sect. A 1975, 79A (6), 713–725. 10.6028/jres.079A.028. PMC6589417 32184525

[ref76] Nikitina V. A.; Kuzovchikov S. M.; Fedotov S. S.; Khasanova N. R.; Abakumov A. M.; Antipov E. V. Effect of the Electrode/Electrolyte Interface Structure on the Potassium-Ion Diffusional and Charge Transfer Rates: Towards a High Voltage Potassium-Ion Battery. Electrochim. Acta 2017, 258, 814–824. 10.1016/j.electacta.2017.11.131.

