# Supplementary material for: Controlling Li Dendritic Growth in Graphite Anodes by Potassium Electrolyte Additives for Li-Ion Batteries
Source: ACS Appl Mater Interfaces. 2022 Sep 12;14(37):42078–92. doi: 10.1021/acsami.2c11175 (PMC9501903; doi:10.1021/acsami.2c11175)
Supplement: Supplementary file 1 — am2c11175_si_001.pdf [file am2c11175_si_001.pdf]

Supporting information

Controlling Li dendritic growth in graphite anodes by potassium electrolyte additives for Li-ion batteries

Sanghamitra Moharana[†], *Geoff West*[†], *Marc Walker*[‡], *Xinjie S Yan*[§], *Melanie Loveridge*[†*]

† Warwick Manufacturing Group (WMG), University of Warwick, Coventry, CV4 7AL, UK.

‡ Department of Physics, University of Warwick, Coventry, CV4 7AL, UK.

§ Impression Technologies Ltd, Unit E Lyons Park, 46 Sayer Dr, Coventry, CV5 9PF, UK.

* Corresponding Author. Email: M.Loveridge@warwick.ac.uk

Table S1. Properties of MagE3 artificial graphite anode and NMC 622 cathode.

	**Artificial Graphite**	**NMC 622**
Electrode Composition	91.83 wt% Hitachi MagE3<br>2 wt% Timcal C45 carbon<br>6 wt% Kureha 9300 PVDF Binder<br>0.17 wt% Oxalic Acid	90 wt% Targray NMC 622<br>5 wt% Timcal C-45<br>5 wt% Solvay 5130 PDVF Binder
Current Collector foil	Copper (Cu)	Aluminium (Al)
Foil Thickness (µm)	10	20
Total Electrode Thickness (µm)	52	58
Total Coating Thickness (µm)	42	38
Calendared Electrode Porosity	30.3%	37.1%
Total Coating loading (mg/cm^2)	6.35	9.78
Total Coating density (g/cm^3)	1.51	2.57
Reversible Capacity @~1C (RT) (mAh/cm^2)	1.92	1.41
Specific Reversible Capacity @~1C (RT) (mAh/g)	330	160

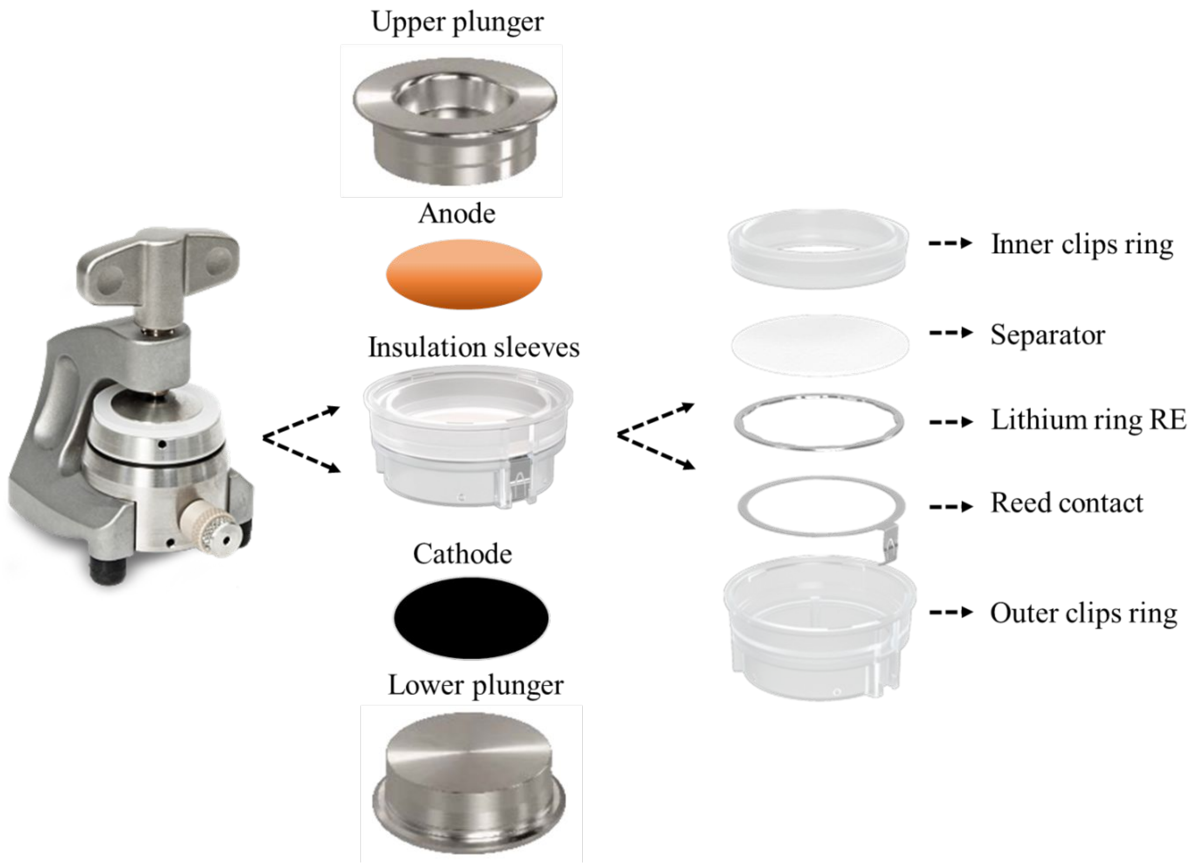

Figure S1. Expanded view of three-electrode ECC-PAT-Core cell components [1].

a)

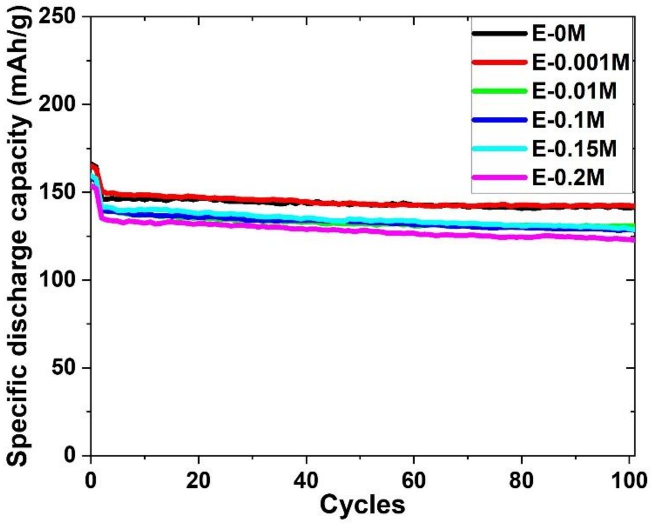

Figure S2. Comparison of specific discharge capacities of graphite | NMC 622 full cell with various electrolytes at 1C (~ 2.8 mA) cycling rate.

E-0M and E-0.001M electrolytes achieve greater specific discharge capacities (~ 150 mAh/g in the 1st cycle and ~140 mAh/g in the 100th cycle) compared to other formulations. A noticeable decrease in discharge capacity is observed as the concentration of KPF_6 additive increases in the electrolyte.

Fast charging develops concentration gradient due to the lack of time availability for the Li^+ ions to intercalate into the graphite layers, which generates heat. This leads to the excessive electrolyte decomposition, henceforth thicker SEI formation on the graphite anode surface. Furthermore, active metallic Li deposition triggers a series of unwanted parasitic reactions, which can be calculated as follows [2,3].

$$\mathbf{k(T,t)} = \frac{\mathbf{1 - CE}}{\mathbf{(time\ of\ one\ cycle)}} \qquad \textbf{Equation-S1}$$

Where, $\mathbf{k(T,t)}$ is parasitic reaction rate, **T** = cell temperature, **t** = calendar time.

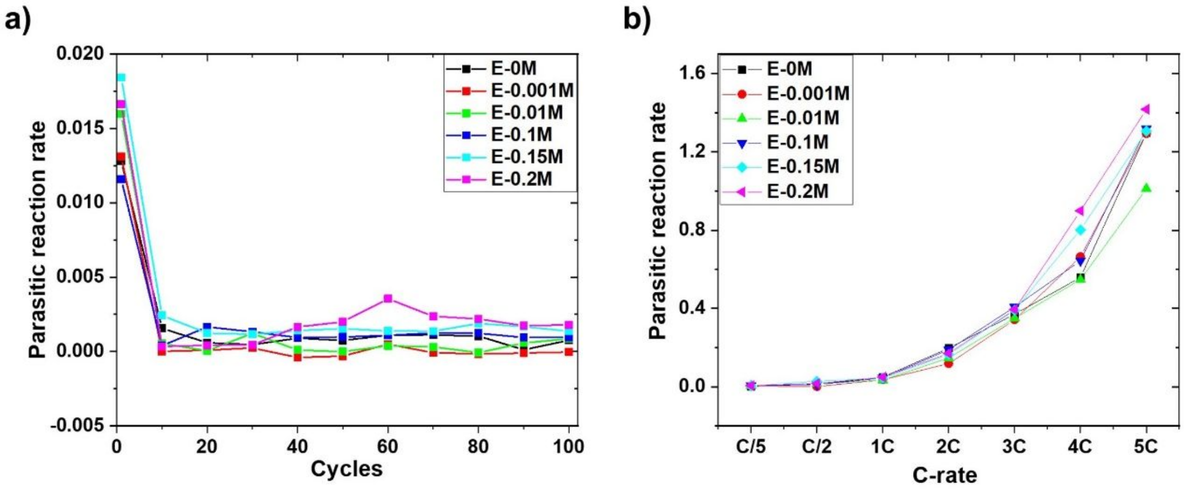

Figure S3. (a) Comparison of parasitic reaction rates at C/2 cycling, (b) 1st cycle average parasitic reaction rate at different C-rates for various electrolytes.

The first cycle parasitic reaction rate is highest because of the SEI formation through electrolyte decomposition. The reaction rate decreases upon successive cycling as SEI stabilises. It is seen that the cells with E-0.15M and E-0.2M electrolytes give rise to higher parasitic reaction rates with cycle numbers as well as C-rates. E-0.001M electrolyte shows the lowest parasitic reaction rates among all the electrolytes at C/2, 2C and 3C rates.

According to Nernst equation, the reduction potential of any electrochemical reaction at 25 °C is as follows.

$$\mathbf{E_{Red} = E^0_{Red} - \frac{RT}{nF} ln \frac{a_{red}}{a_{ox}}}$$

Where, $\mathbf{E_{Red}}$ is the reduction potential at a temperature, $\mathbf{E^0_{Red}}$ is the standard reduction potential, **R** is the universal gas constant (8.314 J K^{-1} mol^{-1}), **T** is the temperature in Kelvin, **n** is the number of the moles of the electrons transferred, **F** is the Faraday constant ($9.648*10^4$ C mol^{-1}), $\mathbf{a_{red}}$ and $\mathbf{a_{ox}}$ is the chemical activity for reduction and oxidation reactions respectively. At lower concentration, chemical activity $\mathbf{a_{red}}$ and $\mathbf{a_{ox}}$ can be simplified to $\mathbf{c_{red}}$ and $\mathbf{c_{ox}}$.

Hence, the equilibrium potential for potassium deposition at 25 °C is as follows,

$$\mathbf{E_{K^+/K} = E^0_{K^+/K} + 0.059 \log a_{K^+}}$$

In LIBs, the Nernst equation with respect to Li/Li^+ becomes,

$$\mathbf{E_{K^+/K} = E^0_{K^+/K} - E^0_{Li^+/Li} + 0.059 \log a_{K^+}}$$

$$\mathbf{E_{K^+/K} = -2.924 - (-3.045) + 0.059 \log a_{K^+}}$$

Where, the reduction potential of Li is -3.045 V and K is -2.924 V. Hence, the final equation is as follows [4],

$$\mathbf{E_{K^+/K} = 0.121 + 0.059 \log a_{K^+}}$$ **Equation-1**

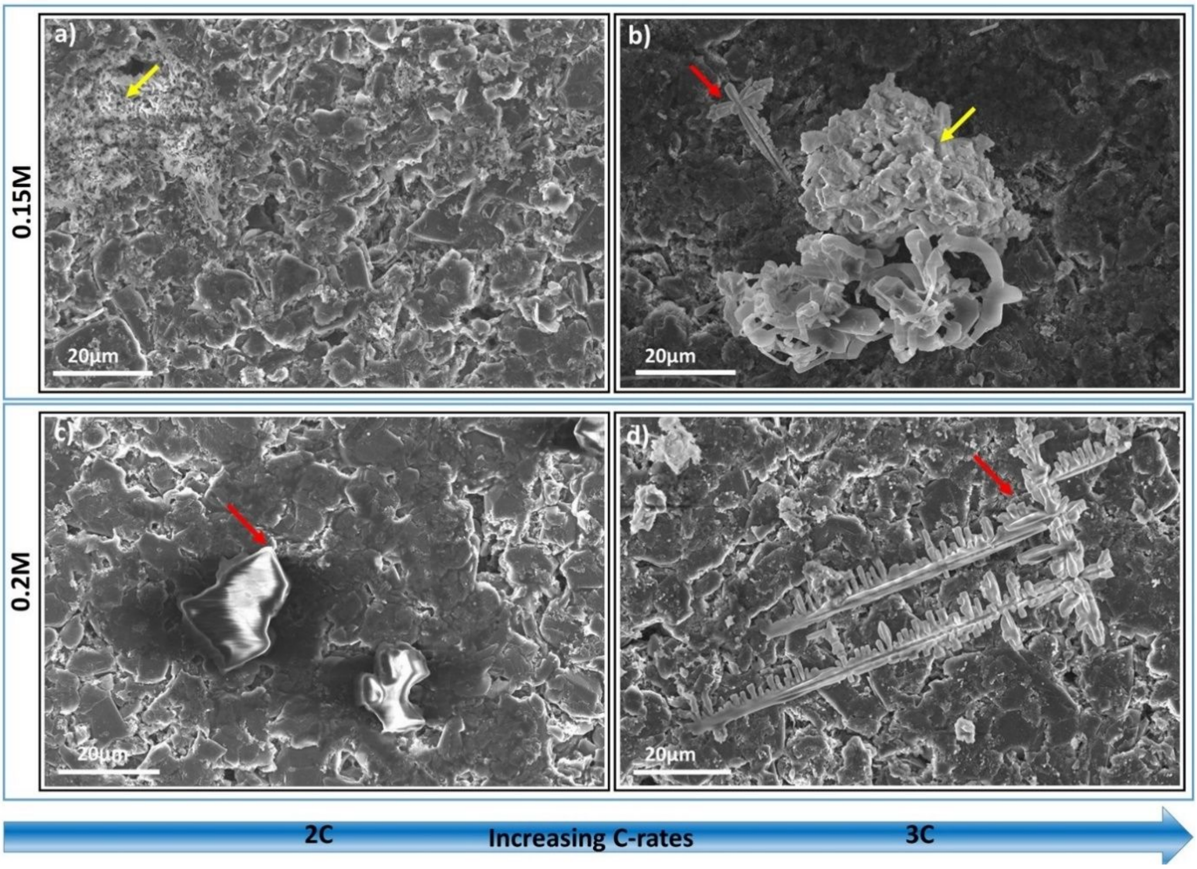

Figure S4. SEM images of graphite cycled with (a, b) E-0.15M and (c, d) E-0.2M at 2C and 3C rate.

Two kinds of deposits with different morphologies are observed in the Figure S4. The cluster of deposits, shown in yellow coloured arrow (Figure S4a and b) are Li dendrites. The second kind of deposit, shown in red coloured arrow, is observed proximal to the Li dendrites. Increase in C rate appears to lengthen and thicken the individual dendritic arms of K, clearly evident in Figure S4d.

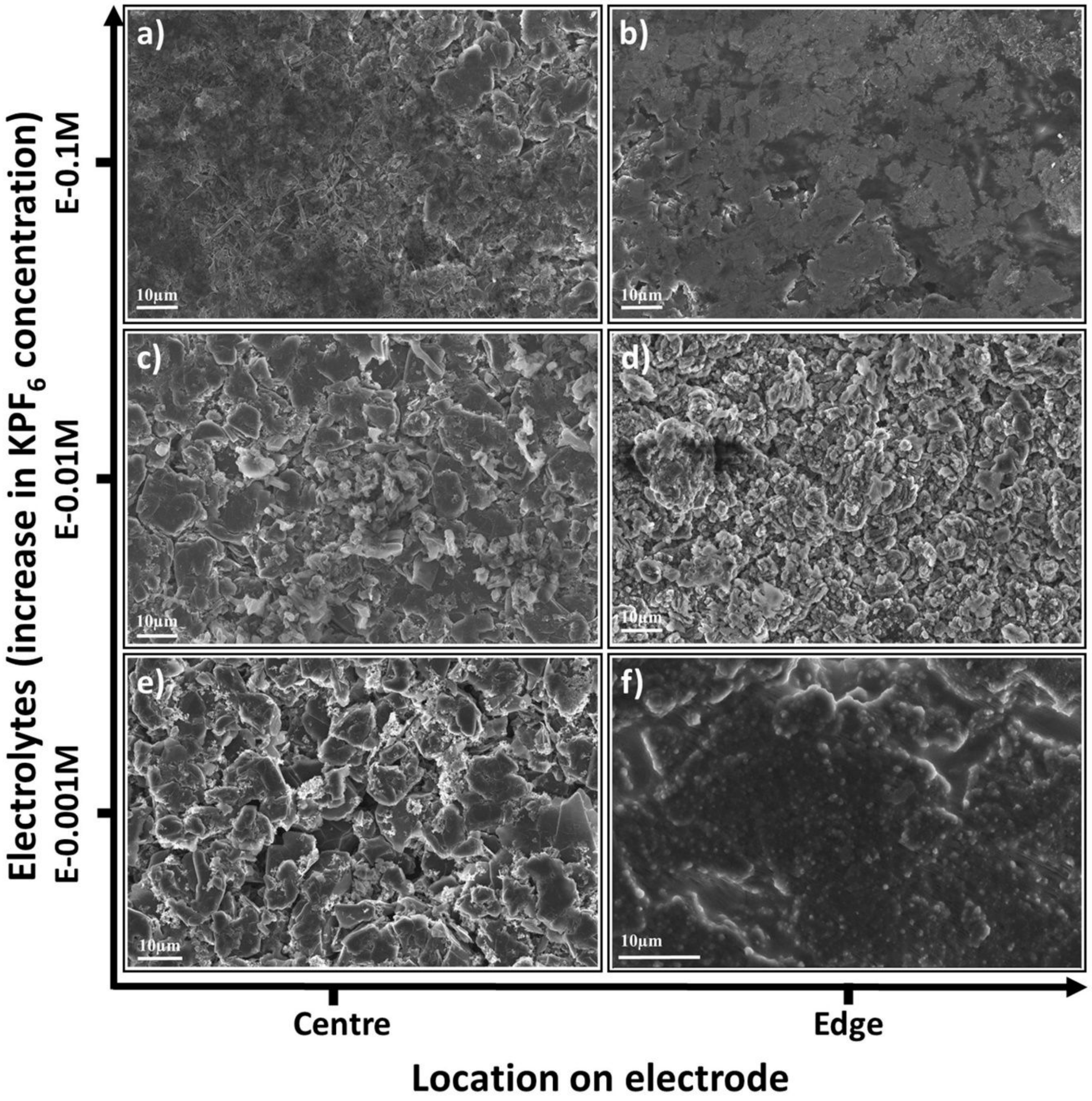

Figure S5. Large area imaging of 2C-cycled graphite morphologies using (a, b) E-0.1M, (c, d) E-0.01M and (e, f) E-0.001M electrolytes at different locations.

Due to the higher surface energy of an electrode's edge, the current density distribution is inhomogeneous, leading to promote Li deposition quicker at the edge, known as 'edge effect' [5]. The severe inhomogeneity in current density distribution triggers Li deposition faster at higher C-rates [6]. No visible dendrites are observed at the edge for all the electrolytes, however heavily covered with film compared to centre of the electrode.

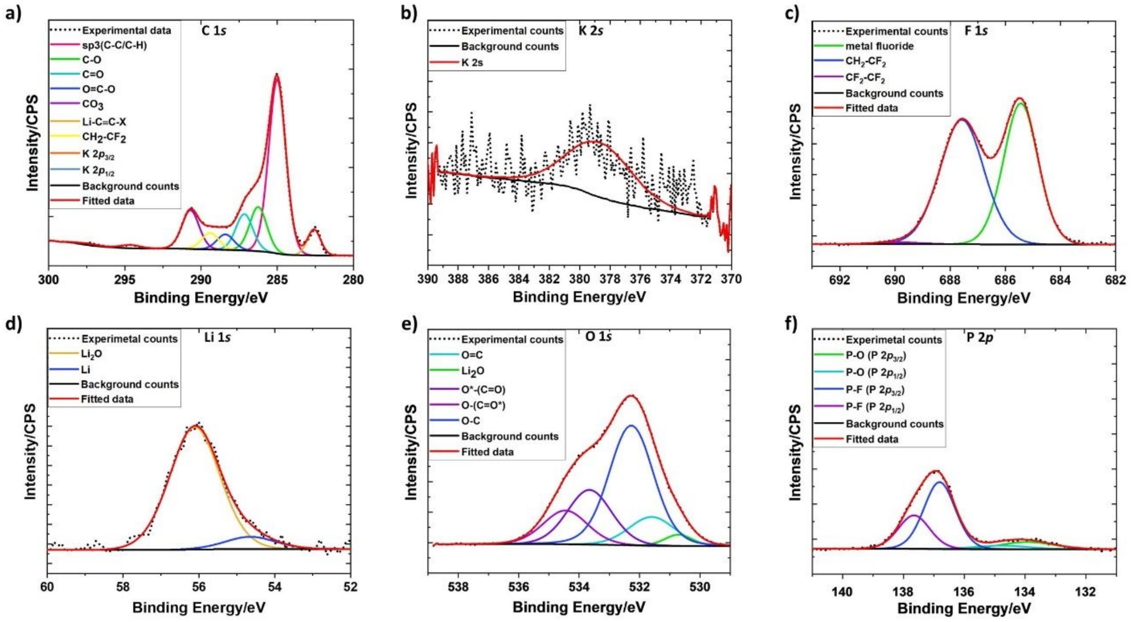

Figure S6. XPS (a) C 1*s*, (b) K 2*s*, (c) F 1*s*, (d) Li 1*s*, (e) O 1*s* and (f) P 2*p* spectra of graphite anode cycled with E-0.1M electrolyte at 2C rate.

The deconvolution of C 1*s* spectrum is similar to that of shown in Figure 7a. An additional component i.e., lithium acetylide (Li-C≡C-X) is detected at ~ 282.5 eV. Lithium acetylide is likely to be formed when metallic Li reacts with SEI compounds, confirming the presence of metallic Li. Furthermore, two tiny peaks are also monitored at ~ 294.6 eV and ~ 297.3 eV towards the end of the C 1s spectrum, which correspond to the K $2p_{3/2}$ and K $2p_{1/2}$. K 2*s* peak at ~ 378.9 eV in Figure S6b ensures that both of these peaks belong to potassium [7].

Table S2. XRF quantification of graphite anode cycled with different electrolytes.

Electrolyte	C-rate	Elements	Concentration
E-0M	C/2	C	96.0178 mass%
		F	3.9748 mass%
		Al	14 ppm
		Si	13 ppm
		P	45 ppm
		K	-
E-0.001M	2C	C	82.7485 mass%
		F	14.2896 mass%
		Al	15 ppm
		Si	27 ppm
		P	2.9366 mass%
		K	210 ppm
E-0.1M	2C	C	81.9692 mass%
		F	13.729 mass%
		Al	21 ppm
		Si	35 ppm
		P	3.4934 mass%
		K	0.8029 mass%
E-0.15M	2C	C	83.7947 mass%
		F	12.4113 mass%
		Al	25 ppm
		Si	59 ppm
		P	2.5133 mass%
		K	1.2722 mass%

Graphite anode using E-0M is used as reference for this study. Al and Si are the common impurities in Cu current collector and the graphite active material, respectively [8,9], that are detected in all the samples in ppm concentrations irrespective of electrolyte and C-rate. The reference sample (E-0M, C/2) has higher C concentration due to slow charging, specifying thinner SEI on graphite. In modified electrolytes, the concentration of F and P increases with decrease in C concentration [10,11] due to higher electrolyte decomposition. As expected, the concentration of K is also increased with incorporation of higher amount of KPF_6 additive.

Table S3. SIMS ion fragments collected from cycled graphite surface.

Mass (amu)	Positive Ion fragments	Mass (amu)	Negative ion fragments
6	^{6}Li	6	^{6}Li
7	^{7}Li	7	^{7}Li
12	C	12	C
13	CH	13	CH
14	N/Li_2	14	N/CH_2
15	CH_3, Li_2H	15	CH_3
16	O	16	O
17	OH	17	OH
19	F from $LiPF_6$/LiF	19	F
23	LiO	22	^{6}LiO
24	LiOH	23	LiO
26	C_2H_2	24	LiOH, C_2
27	C_2H_3	25	C_2H
28	Si	26	C_2H_2, CN
29	C_2H_5, COH	28	Si, CO
30	Li_2O	31	OCH_3, P, CF
31	Li_2OH, CF, C_2H_7	32	O_2, S
**32**	**$^{6}Li^{7}LiF$**, O_2	33	SH
**33**	**Li_2F**	35	OF
37	Li_3O	36	C_3
39	LiO_2/^{39}K	37	C_3H, H_2OF
40	Li_3F	38	F_2
41	C_3H_5, H_3F_2 From PVDF/^{41}K	39	F_2H
43	C_3H_7, C3H3O From PVDF	40	C_2O, LiO_2H
45	C_2H_5O	41	C_2HO, Li $(OH)_2$
46	Li_3N, Li_2O_2	42	CNO, C_2H_2O
51	C_4H_3, CHF_2 From PVDF	43	$C_2H_3O_2$, C_2F
**52**	**Li_2F_2**	**44**	CO_2, **$^{6}LiF_2$**
53	C_2H_6OLi	**45**	**LiF_2**, CO_2H

55	C_4H_7	47	PO
57	C_4H_9	48	C_4, SO, $(LiOH)_2$
**58**	**$^6Li^7Li_2F_2$**	49	C_4H
**59**	**Li_3F_2**	50	CF_2
63	C_4H_8Li	54	LiPO
67	Li_5O_2	55	C_2P, LiO_3
69	Ga	56	LiO_3H
71	LiO_4	57	CF_3
74	Li_2CO_3	58	Li $(OH)_3$
77	$C_2H_5O_3$, $C_3H_3F_2$ From PVDF	59	$C2H_3O_2$
81	Li_3CO_3	60	CO_3
85	Li_4F_3	61	HCO_3
89	EC+H	63	PO_2
93	Li (VC)	64	HPO_2, LiF_3, SO_2
95	Li (EC), $C_2H_2F_3$ from PVDF	65	$Li_2(OH)_3$
97	$C_5H_5O_2$	66	C_3NO
99	$C_5H_7O_2$	67	$LiCO_3$
102	Li_2(EC)	70	$LiPO_2$
103	EC+CH_3	**71**	**Li_2F_3**, LiO_4
105	EMC+H	72	C_6
109	Li_3(EC)	74	C_5N
111	Li (EMC)	79	PO_3
118	Li_2(EMC)	80	SO_3
125	Li_3(EMC)	81	SO_3H
127	$C_2H_2O_5Li_3$	82	PFO_2
129	EC+C_3H_5	85	POF_2
131	EC+C_2H_3O	87	EC-H/$C_3H_3O_3$
159	Li_2+PF_6	88	Li_3F
		89	$Li_3(OH)_4$
		95	PO_4
		96	SO_4
		97	H_2PO_4

		100	SO_4
		101	PO_2F_2, $C_3H_2O_2P$
		102	$LiPO_4$
		103	EMC-H
		105	EC+OH
		113	$Li_4(OH)_5$
		121	EMC+OH
		123	$C_2H_4PO_4$
		127	Li $(CO_3)_2$
		131	EC+C_2H_3O
		137	$Li_5(OH)_6$, $C_3H_6PO_4$
		145	PF_6
		147	EMC+C_2H_3O
		149	CF_3SO_3

The ion species associated with metal fluoride are highlighted in green and bold letters in the above Table S3.

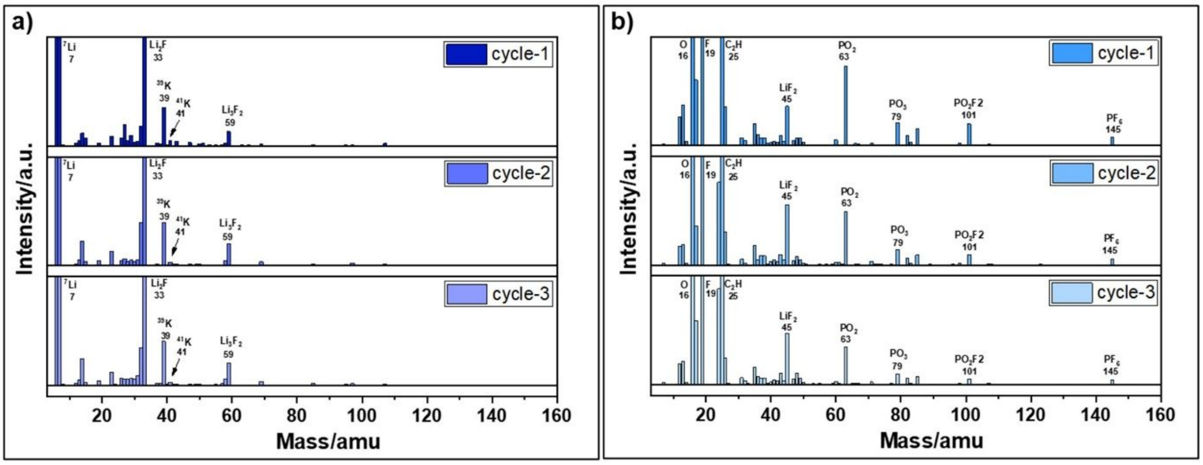

Figure S7. SIMS (a) positive and (b) negative ion mode mass spectra for number of cycles at same locations on cycled graphite using E-0.1M electrolyte.

The ratio of peaks at 97 amu/99 amu in positive ion and 155amu/157 amu in negative ion mode do not match with the abundance ratio of $^{39}K^{+}/^{41}K^{+}$, which depicts the absence of KF in SEI layer in each cycle.

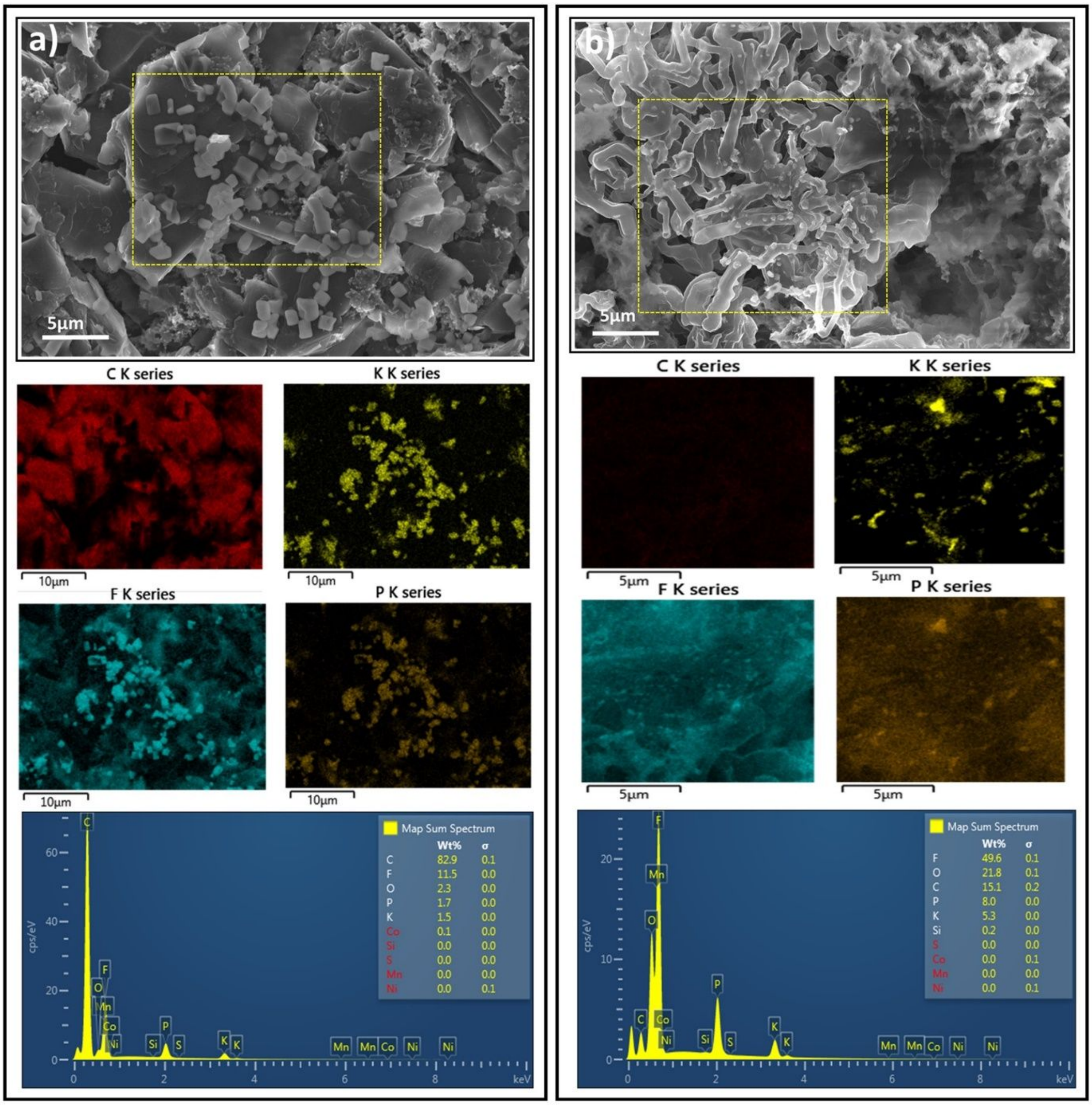

Figure S8. SEM images, corresponding EDX maps and spectra presenting the preferential deposition of potassium on graphite anode using E-0.1M at 4C rate.

REFERENCES

(1) Equipment, E.-C. test. *EL- CELL electrochemical test equipment*. https://el-cell.com/products/test-cells/standard-test-cells/pat-cell/.

(2) Smith, A. J.; Burns, J. C.; Dahn, J. R. A High Precision Study of the Coulombic Efficiency of Li-Ion Batteries. *Electrochem. Solid-State Lett.* **2010**, *13* (12). https://doi.org/10.1149/1.3487637.

(3) Madani, S. S.; Schaltz, E.; Kær, S. K. Effect of Current Rate and Prior Cycling on the Coulombic Efficiency of a Lithium-Ion Battery. *Batteries* **2019**, *5* (3). https://doi.org/10.3390/batteries5030057.

(4) Komaba, S.; Itabashi, T.; Kimura, T.; Groult, H.; Kumagai, N. Opposite Influences of K+ versus Na+ Ions as Electrolyte Additives on Graphite Electrode Performance. In *Journal of Power Sources*; 2005; Vol. 146, pp 166–170. https://doi.org/10.1016/j.jpowsour.2005.03.121.

(5) Smyrl, W. H.; Newman, J. Current Distribution at Electrode Edges at High Current Densities. *J. Electrochem. Soc.* **1989**, *136* (1), 132–139. https://doi.org/10.1149/1.2096572.

(6) Birkenmaier, C.; Bitzer, B.; Harzheim, M.; Hintennach, A.; Schleid, T. Lithium Plating on Graphite Negative Electrodes: Innovative Qualitative and Quantitative Investigation Methods. *J. Electrochem. Soc.* **2015**, *162* (14), A2646–A2650. https://doi.org/10.1149/2.0451514jes.

(7) Biesinger, M. C. *X-Ray Photoelectron Spectroscopy Reference Pages*. http://www.xpsfitting.com/2020/02/potassium.html.

(8) Murdock, A. T.; van Engers, C. D.; Britton, J.; Babenko, V.; Meysami, S. S.; Bishop, H.; Crossley, A.; Koos, A. A.; Grobert, N. Targeted Removal of Copper Foil Surface Impurities for Improved Synthesis of CVD Graphene. *Carbon N. Y.* **2017**, *122*, 207–216. https://doi.org/10.1016/j.carbon.2017.06.075.

(9) Inagaki, M.; Noda, T. Distribution of Inorganic Impurities in Graphite Electrodes. *Bull. Chem. Soc. Jpn.* **1963**, *36* (1), 112–114. https://doi.org/10.1246/bcsj.36.112.

(10) Mussa, A. S.; Liivat, A.; Marzano, F.; Klett, M.; Philippe, B.; Tengstedt, C.; Lindbergh, G.; Edström, K.; Lindström, R. W.; Svens, P. Fast-Charging Effects on Ageing for Energy-Optimized Automotive LiNi1/3Mn1/3Co1/3O2/Graphite Prismatic Lithium-Ion Cells. *J. Power Sources* **2019**, *422*, 175–184. https://doi.org/10.1016/j.jpowsour.2019.02.095.

(11) Yang, X. G.; Liu, T.; Gao, Y.; Ge, S.; Leng, Y.; Wang, D.; Wang, C. Y. Asymmetric Temperature Modulation for Extreme Fast Charging of Lithium-Ion Batteries. *Joule* **2019**, *3* (12), 3002–3019. https://doi.org/10.1016/j.joule.2019.09.021.
